# Identification of a master regulator Msd1 that governs meiotic entry in a global basidiomycete pathogen

**DOI:** 10.1073/pnas.2536339123

**Published:** 2026-06-24

**Authors:** Fanglin Zheng, Yanli Cao, Huiting Chen, Lili Yan, Feng Lv, Yuan Huang, Man Chen, Lin Su, Zhuozhuo Liu, Ye Huang, Tuyetnhu Pham, Xinping Xu, Xiaorong Lin

**Affiliations:** ^a^ Jiangxi Provincial Key Laboratory of Respiratory Diseases, Jiangxi Institute of Respiratory Diseases, The Department of Respiratory and Critical Care Medicine, The First Affiliated Hospital, Jiangxi Medical College, Nanchang University, Nanchang, Jiangxi 330006, China; ^b^Jiangxi Clinical Research Center for Respiratory Diseases, Nanchang, Jiangxi 330006, China; ^c^ Jiangxi Hospital of China-Japan Friendship Hospital, National Regional Center for Respiratory Medicine, Nanchang, Jiangxi 330209, China; ^d^Department of Microbiology, University of Georgia, Athens, GA 30602; ^e^ Department of Immunology, School of Medicine, Jiangxi Medical College, Nanchang University, Nanchang, Jiangxi 330006, China; ^f^ Department of Geriatric Medicine, The First Affiliated Hospital, Jiangxi Medical College, Nanchang University, Nanchang, Jiangxi 330006, China; ^g^Department of Plant Biology, University of Georgia, Athens, GA 30602

**Keywords:** meiosis, sexual reproduction, *Cryptococcus*, Msd1, sporogenesis

## Abstract

Meiosis is a hallmark of sexual reproduction. Although the core meiotic machinery is evolutionarily conserved from unicellular yeasts to Metazoan, the key regulators responsible for initiating meiosis vary across species and remain largely unidentified in major eukaryotic lineages, including the major phylum Basidiomycota in the Kingdom Fungi. The basidiomycete fungus *Cryptococcus neoformans* is a critical pathogen listed by the World Health Organization that causes life-threatening meningoencephalitis worldwide. Meiosis not only indirectly facilitates cryptococcal pathogenicity through generating genetically diverse spores but also directly contributes to host adaptation and disease progression. Here, we identified a novel transcription factor Msd1 that is responsible for activating meiosis in *C. neoformans*. Deletion of *MSD1* impaired multiple sexual development events and strikingly abolished meiosis and subsequent sporogenesis (gametogenesis). Conversely, overexpression of *MSD1* alone is sufficient to drive meiosis and the formation of meiotic spores, even in the absence of the external mating-inducing cues or when the mating pathway is genetically inactivated. Mechanistically, we demonstrated that Msd1 initiates meiotic entry by targeting two interconnected pathways. First, Msd1 activates the transcription of multiple evolutionarily conserved core meiosis-specific genes such as meiotic recombinase Dmc1. Second, Msd1 activates two RNA-binding proteins, Csa1 and Csa2, which ensure spatiotemporal expression of the aforementioned meiosis-specific genes to drive meiosis and sporogenesis. Collectively, our findings demonstrate that meiosis and the sequential sexual development events are spatiotemporally orchestrated by the master regulator Msd1, and is the first reported regulator initiating meiotic entry in a basidiomycete fungus.

## Significance

Meiosis is indispensable for the generation of genetically diverse gametes in sexually reproducing organisms. In the pathogenic *Cryptococcus* species complex, extensive meiotic recombination occurred during sexual development, facilitating the emergence of hypervirulent and drug-resistant variants. Despite the importance of meiosis in driving cryptococcal virulence evolution, the underlying molecular mechanism that regulates meiotic entry remains poorly understood in this fungal pathogen. In this study, we found that meiotic entry and sporogenesis are governed by a master transcription factor Msd1 in *Cryptococcus neoformans*. We demonstrated that Msd1 initiates meiosis through activating a dual regulatory circuit that controls the spatiotemporal expression of core meiotic genes. These findings provided strong evidence toward a comprehensive understanding of meiosis initiation in a basidiomycete.

Sexual reproduction is ubiquitous across eukaryotes. It typically involves gametic fusion that doubles the chromosomes, followed by meiosis to generate four meiotic gametes. Meiosis is a specialized form of cell division characterized by one round of genomic DNA replication followed by two successive chromosome segregations, resulting in a reduction in nuclear DNA content and cellular ploidy. During meiosis, extensive chromosome recombination occurs, generating genetically diverse gametes and facilitating the elimination of deleterious mutations, thus providing a critical evolutionary force for eukaryotic organisms. Commitment to meiosis is a highly coordinated and tightly regulated process (1, 2). Although the core meiotic machinery is evolutionarily conserved from yeast to vertebrates, the signals and the key regulators that govern meiotic entry vary across species (1, 3).

Meiosis is typically initiated in response to integrated external and intrinsic cues, and its entry is governed at the transcriptional and/or the posttranscriptional level by key regulators. In the model ascomycete budding yeast *Saccharomyces cerevisiae*, the transcriptional factor (TF) Ime1 controls meiosis initiation (4, 5). *IME1* is repressed in haploid cells and activated in diploid cells under appropriate conditions (4, 6). Once expressed, Ime1 interacts with Ume6 and converts this transcriptional repressor into an activator to trigger the expression of the early meiosis genes (EMGs) (7, 8). The EMGs promote the early meiosis events during meiotic interphase and prophase I, including premeiotic DNA replication, pairing of homologous chromosomes, synapsis, and homologous recombination (9, 10). Thus, Ime1 in budding yeast functions as a molecular switch for entry into meiosis. In the fission yeast *Schizosaccharomyces pombe*, an RNA-binding protein Mei2 dictates initiation of meiosis (11). *MEI2* expression is induced by the transcription factor Ste11 in response to nitrogen starvation (12). In diploid cells, the unphosphorylated Mei2 stabilizes meiosis-specific transcripts, thereby ensuring the expression of meiotic genes and entry into meiosis (13). During mitotic growth in haploid cells, Mei2 activity is suppressed through phosphorylation by the kinase Pat1 (14), thus preventing the entry into meiosis. Basidiomycetes, including rust fungi and mushroom species, diverged from ascomycetes from a common ancestor about 600 Mya and constitute a major phylum within the Kingdom Fungi (15). Because no master meiotic activator has been identified in this phylum, it remains unclear whether basidiomycetes employ similar regulatory mechanisms governing the initiation of meiosis as those observed in the model ascomycetes or have evolved distinct regulatory pathways.

The basidiomycete *Cryptococcus neoformans* is ranked as a top critical fungal pathogen by the WHO (16, 17). This fungus causes cryptococcal meningoencephalitis, a fatal disease that is responsible for ~19% of deaths in AIDS patients (18). Due to its well-characterized sexual life cycle and associated features that resemble those in higher eukaryotes (such as uniparental mitochondrial DNA inheritance and large sex determining genetic region), *C. neoformans* has been increasingly recognized as a model organism for studying sexual reproduction and meiosis (19, 20). Over the past three decades, studies have demonstrated that meiosis and/or para-meiotic processes indirectly and directly promote cryptococcal adaptation to the host and virulence. For instance, sexual reproduction enables the emergence of hypervirulent or drug-resistant isolates through de novo mutations and extensive recombination through meiosis (21–25). In addition, meiosis is also required for the production of stress-resilient infectious spores (26, 27). Furthermore, our previous work reveals that core meiotic genes in *C. neoformans* are activated during infection, which contributes to cryptococcal ploidy reduction and facilitates the generation of antigenotoxic progenies and adaptation to the hostile host condition (28). Despite the documented importance of meiosis for cryptococcal evolution and host adaptation, the key regulator(s) that governs the meiotic entry remains unknown. The goal of this study is to identify the master meiosis activator in this basidiomycete.

## 1. Results

### 1.1. Identification of Msd1 as a Regulator of the Morphogenesis Factor Znf2 Via Yeast One-Hybrid (Y1H) Screen.

**Fig. 1.**
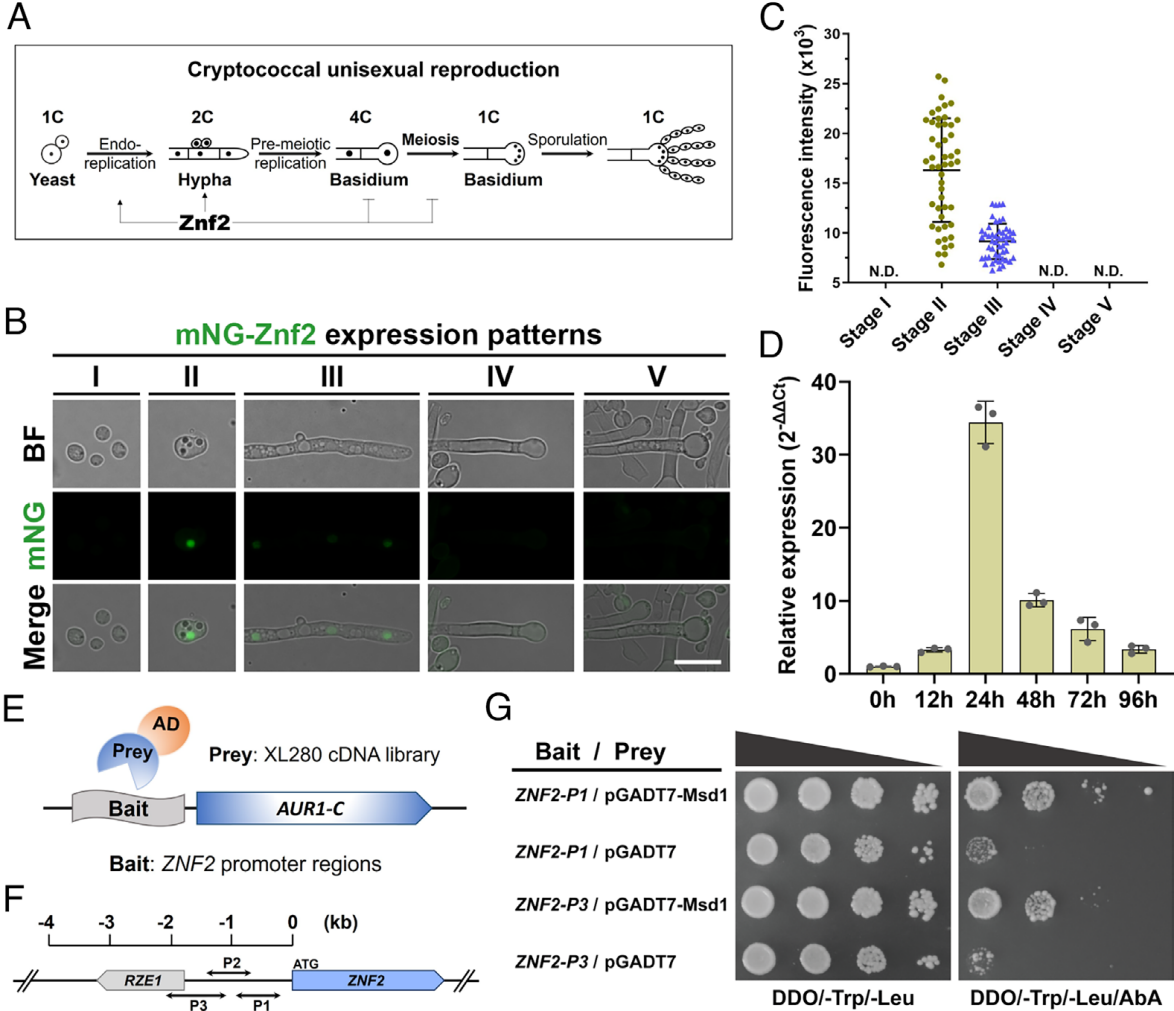
Identification of a novel TF, Msd1, that binds to the *ZNF2* promoter via a Y1H screen. (*A*) Schematic representation of the cellular morphology and ploidy dynamics during the cryptococcal unisexual reproduction. *1C*, *2C*, and *4C* represent the haploid, diploid, and tetraploid states of the genome, respectively. (*B*) The expression pattern of mNeonGreen (mNG)-Znf2 during different stages of unisexual development. (Scale bar, 10 μm.) (*C*) Quantification of the mNG-Znf2 fluorescence intensity across the five unisexual developmental stages as indicated in panel *B*. N.D., not detected. (*D*) RT-qPCR analysis of the transcriptional dynamics of *ZNF2* during the different time points of unisexual development. Error bars represent mean ± SD from three biological replicates. (*E*) Schematic diagram of the Y1H screen system used in this study. (*F*) Schematic representation of the three segments of the 2.5-kb promoter region of *ZNF2* designed for Y1H screen. (*G*) The confirmative Y1H assay verified the binding of Msd1 to *ZNF2*’s P1 and P3 promoter regions.

The cryptococcal sexual cycle is a highly integrated process that necessitates the precise coordination of dynamic ploidy changes with a series of morphological developmental events (Fig. 1*A*). *C*. *neoformans* can undergo either **a**-α bisexual reproduction or unisexual reproduction. Due to the predominance of α isolates (>99%), unisexual reproduction is considered the dominant sex form occurring in nature (29, 30). During unisexual reproduction, nuclear diploidization is typically achieved through endoreplication, followed by premeiotic DNA replication and meiosis, which occurs concurrently with basidial differentiation at the hyphal apex, ultimately leading to the generation of haploid basidiospores (20) (Fig. 1*A*). We divided unisexual development into five stages based on the cellular differentiation characteristics: yeast (Stage I), germ-tube (undergoing yeast-hypha transition, Stage II), hypha (Stage III), basidium (Stage IV), and basidium with sporulation (Stage V) (*SI Appendix*, Fig. S1*A*). The early stage of sexual reproduction, including mating response and conjugation, is controlled by the HMG domain transcription factor Mat2, whereas the yeast-hypha transition and hyphal extension is governed by the C2H2-type zinc finger transcription factor Znf2 (31, 32). The potential involvement of Znf2 in the later stages of sexual development, such as basidium formation, meiosis, and sporulation, have yet to be elucidated.

To address this question, we generated a knock-in strain *mNG*-*ZNF2*^KI^ where *ZNF2* was tagged with mNG at its native locus (*SI Appendix*, Fig. S1 *B* and *C*). This *mNG*-*ZNF2*^KI^ strain behaved as the wild-type (WT) strain under mating-inducing condition (*SI Appendix*, Fig. S1*D*), indicating that mNG-Znf2 is functionally equivalent of the untagged native Znf2. During unisexual development, the mNG-Znf2 signal was undetectable in stage I yeast cells, became prominent in stage II cells undergoing the yeast-to-hypha transition, and persisted in stage III hyphal cells (Fig. 1 *B* and *C*). However, once hypha tips differentiated further into stage IV (basidia) and stage V (sporulation), its signal was no longer detectable (Fig. 1 *B* and *C*). Consistent with the mNG-Znf2 expression pattern, the transcript level of *ZNF2* peaked at 24 h (early hyphal stage) and subsequently decreased throughout the middle and late stages of unisexual development (Fig. 1*D*). Both the transcript and the protein expression pattern align with the role of Znf2 as the master transcriptional activator of yeast-hypha morphogenesis during the earlier stages of sexual development (32). It is possible that *ZNF2* may need to be turned off to permit the progression into the later stages of sexual development. Consistent with this idea, forced constitutive expression of *ZNF2* suppressed sporulation (*SI Appendix*, Fig. S2), as we also noticed previously (32, 33). These observations raise the possibility that a different regulator is responsible for activating meiosis during the later stages of sexual development.

As each wave of key transcription factors may control, and be regulated by, those preceding or succeeding it, we hypothesize that the transcription factor responsible for initiating meiosis, if exists, could be linked to Znf2. To investigate this possibility, we employed a Y1H screening system to identify potential regulators that bind to the *ZNF2* promoter by using a cryptococcal cDNA library prepared from strain XL280 grown under multiple mating-inducing conditions (Fig. 1*E*). A 2.5 kb upstream region of the *ZNF2* coding sequence was divided into three fragments (P1, P2, and P3) and used as baits by cloning them upstream of the *AUR1-C* reporter gene (Fig. 1*F*), which encodes a point-mutated inositol phosphoryl ceramide synthase that confers resistance to Aureobasidin A (AbA). Among these three baits, the P2 region was excluded from subsequent experiments due to autoactivation in yeast. After multiple rounds of screening using the yeast strains carrying P1 and P3 baits, 10 potential DNA-binding proteins were identified (*SI Appendix*, Table S1). For these hits, we generated knock-out cryptococcal strains individually and performed phenotypic analysis under mating-inducing condition (*SI Appendix*, Fig. S3*A*). We focused on *CNA00260* as its deletion caused the most dramatic reduction in filamentation on V8 medium (*SI Appendix*, Fig. S3*A*), and complementation with a WT copy at its native locus restored filamentation (*SI Appendix*, Fig. S3 *B* and *C*). Moreover, overexpression of *CNA00260* drove a constitutive yeast-hyphal transition under mating-suppressing condition (*SI Appendix*, Fig. S3*D*). We named this gene *MSD1* (Meiosis and Sexual Development regulator 1) based on subsequent studies.

The *MSD1*-encoded protein contains a centrally located GATA-type zinc finger domain (amino acids 256 to 306, E-value = 1.70e-07) (*SI Appendix*, Fig. S4*A*). Its orthologs are exclusively found in the order Tremellales in Basidiomycota. Based on phylogenetic analysis (*SI Appendix*, Fig. S4*B*), they are well separated from the branch of the widely distributed orthologs of Gat1, the closest homolog of Msd1 in *C. neoformans*, across Ascomycetes and Basidiomycetes, indicating that Msd1 is a Tremellales-specific transcription factor. Confirmative Y1H assay verified the binding of this transcription factor to the two *ZNF2* promoter regions (Fig. 1*G*). Deletion of the *MSD1* gene elevated the transcript level of *ZNF2*, while its overexpression reduced *ZNF2* transcript level compared to that in the WT when incubated on V8 medium for 24 h (*SI Appendix*, Fig. S5 *A* and *B*). Moreover, mNG-Znf2 signal was significantly increased in the *msd1*Δ mutant compared to the parental *mNG*-*ZNF2*^KI^ strain under the same condition (*SI Appendix*, Fig. S5 *C* and *D*). These findings indicate that Msd1 plays a repressive role in regulating *ZNF2* expression during the early sexual development stage. Interestingly, *MSD1* was also identified as a direct downstream target of Znf2 based on our previous ChIP-seq and ATAC-Seq data (34), implying a potential interregulation between Msd1 and Znf2. Collectively, these results indicated that Msd1 is a novel regulator of the morphogenesis factor Znf2.

### 1.2. Msd1 Is Required for Activating Meiosis, and It Directly Regulates Meiotic Gene Expression.

**Fig. 2.**
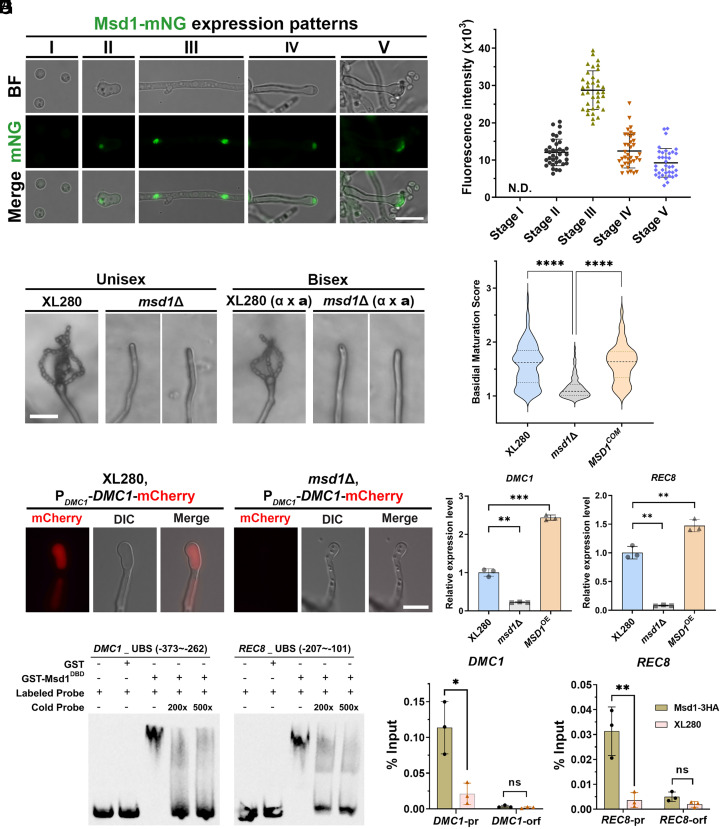
Msd1 activates meiosis genes and is crucial for sporulation. (*A*) Expression pattern of Msd1-mNG during the different stages of unisexual reproduction. BF and mNG indicate brightfield and mNeonGreen, respectively. (*B*) Measurement of the Msd1-mNG fluorescence intensity at different stages during unisexual reproduction as in panel *A* (n = 36). N.D., not detected. (*C*) Sporulation assay of the XL280 and the *msd1*Δ mutant during unisexual and bisexual reproduction. (Scale bar, 20 μm.) (*D*) Basidial maturation score assay of the WT, *msd1*Δ, and *MSD1*^com^ strains during unisexual reproduction on V8 medium for 2 wk. BMS evaluates the maturation level of the basidium by calculating the ratio of the diameter of the basidium to that of the connected hypha. ****P < 0.0001, two-tailed Student’s *t* test. (*E*) Expression of Dmc1-mCherry in the WT and the *msd1*Δ strains during unisexual reproduction on V8 medium. (Scale bar, 10 μm.) (*F*) RT-qPCR assay of the relative transcript levels of *DMC1* and *REC8* in WT, *msd1*Δ, and *MSD1*^OE^ strains on V8 medium for 24 h. Error bars: mean ± SD; Statistical significance was determined by two-tailed Student’s *t* test (***P < 0.001, **P < 0.01). (*G*) Msd1 binds to the promoter regions of *DMC1* and *REC8* containing the upstream binding site (UBS) of the GATA-type TF as demonstrated by EMSA. (*H*) ChIP-qPCR assay demonstrating in vivo binding of Msd1-3HA to the promoter region of *DMC1* and *REC8*, but not to their ORFs. Error bar indicates mean ± SD from three biological replicates. Statistical significance was determined by two-tailed Student’s *t* test (**P < 0.01; *P < 0.05; ns, *P* > 0.05, not significant).

To examine the expression dynamics of Msd1, we generated a C-terminal mNG tagged Msd1 knock-in strain (*MSD1*-*mNG*^KI^) (*SI Appendix*, Fig. S6 *A* and *B*). The Msd1-mNG fusion protein was functional based on its ability to support filamentation on V8 medium comparably to WT (*SI Appendix*, Fig. S6*C*). The Msd1-mNG signal was barely detectable in the yeast cell stage, appeared in germ tubes (Stage II) and reached the highest expression level in the hypha growth stage (Stage III) (Fig. 2 *A* and *B*). Unlike Znf2, Msd1-mNG signal remained strong even in the later stages of sexual development, including the nascent and mature basidium (Fig. 2 *A* and *B*). At all of these expressed stages, Msd1-mNG signal showed colocalization with the nuclear staining signal (*SI Appendix*, Fig. S6*D*). Thus, Msd1 is expressed and localized to the nucleus throughout the entire sexual reproduction cycle, exhibiting a much broader temporal coverage than Znf2.

Given that Msd1 is expressed throughout the entire sexual cycle, including the late stages of sexual development, we wondered if it plays a role in basidial differentiation, meiosis, and sporulation. We noticed that the sparse hyphae derived from the *msd1*Δ mutant during either unisexual or bisexual cycles failed to sporulate (Fig. 2*C*), even after extended incubation. Basidial maturation is tightly coordinated with meiosis, preceding the generation of long chains of spores (Fig. 1*A*) (35). We found that deletion of *MSD1* resulted in a dramatically decreased basidial maturation score (BMS) (Fig. 2*D*), with less than 6.45% of basidia meeting the maturation threshold (BMS > 1.6) (35), while complementation of *MSD1* restored the BMS to the WT level (Fig. 2*D*). Therefore, Msd1 is required for basidial maturation during cryptococcal sexual development.

Since meiosis is a prerequisite of sporulation, we next investigated whether Msd1 is required for meiosis. To this end, we monitored the expression of the mCherry tagged Dmc1, a highly conserved meiosis-specific recombinase (29, 36). Dmc1-mCherry was exclusively expressed within the basidium in WT (Fig. 2*E*), as we reported previously (33, 36). Notably, we could no longer detect any Dmc1-mCherry signal in hyphal apexes once *MSD1* was deleted (Fig. 2*E*). At the transcript level, *DMC1* was decreased by 77.8% in the *msd1*Δ mutant and increased by 243% in the *MSD1* overexpression strain (*MSD1*^OE^) (Fig. 2*F*). Similarly, the transcript level of *REC8*, a highly conserved meiotic cohesion complex subunit gene, was reduced by 91.7% in *msd1*Δ and increased by 46.9% in the *MSD1*^OE^ strain (Fig. 2*F*). Collectively, these observations indicate that Msd1 plays a crucial role in the activation of the core meiosis-specific genes.

We then tested if Msd1 directly activates the expression of these meiotic specific genes by performing an electrophoretic mobility shift assay (EMSA). To this end, we purified glutathione S-transferase (GST) and GST-Msd1^DBD^ (GST tagged DNA-binding domain of Msd1) recombinant proteins from *Escherichia coli* (*SI Appendix*, Fig. S7). These recombinant proteins were subsequently incubated with biotin-labeled DNA fragments corresponding to the *DMC1* and the *REC8* promoter regions, which contain the putative binding motif HGATAR (H = A, C, T, and R = A, G) of the GATA-type family transcription factor (37). As shown in Fig. 2*G*, a prominent band with a much higher molecular weight was detected when the GST-Msd1^DBD^ fusion protein, but not the GST control protein, was incubated with the biotin-labeled *DMC1* and *REC8* probes, while this upshifted band disappeared when excess unlabeled (cold) probe was added to the reaction. This result indicates a specific binding of Msd1 to the *DMC1* and *REC8* promoters in vitro. Direct in vivo binding of Msd1 to the *DMC1* and *REC8* promoter region, but not their open reading frame (ORF), was further confirmed by ChIP-qPCR assay using a C terminal 3 × HA tagged Msd1 recombinant strain (Fig. 2*H*). Taken together, these results demonstrate that Msd1 directly activates the expression of the two well-defined core meiotic genes *DMC1* and *REC8*, and it is crucial for the commitment to meiosis during cryptococcal sexual development.

### 1.3. Msd1 Is Required for Initiating Meiosis and Sporulation in the Diploid Background.

During sexual reproduction, diploidization occurs initially, followed by premeiotic replication and two reductive meiotic divisions. Diploidization is achieved either through syngamy (cell–cell fusion) in bisexual reproduction or through mostly endoreplication during unisexual reproduction in *C. neoformans* (Fig. 1*A*) (20, 38). As endoreplication primarily occurs prior to hyphal growth, blastospores budded laterally from the hyphae are typically diploid during unisexual reproduction (Fig. 1*A*) (29). To examine if Msd1 is required for diploidization, we dissected the blastospores from the WT and the *msd1*Δ mutant cultured on V8 medium and examined their ploidy through PI staining and flow cytometry analysis (*SI Appendix*, Fig. S8*A*). Expectedly, 80% and 75% of blastospores isolated from XL280 and the *MSD1* complemental strain (*MSD1*^com^) were diploid (*SI Appendix*, Fig. S8*B*). In contrast, all blastospores dissected from the *msd1*Δ mutant were haploid (*SI Appendix*, Fig. S8*B*), indicating that Msd1 is required for diploidization during unisexual reproduction.

To analyze if Msd1 is required exclusively for diploidization prior to meiosis or whether it is also required for meiosis even after diploidization is achieved, we generated a diploid *msd1*Δα/*msd1*Δ**a** mutant strain through **a-**α cell fusion (*SI Appendix*, Fig. S8*C*). The ploidy state of the wildtype XL280 α/**a** and the *msd1*Δα/*msd1*Δ**a** strains were confirmed by flow cytometry assay (*SI Appendix*, Fig. S8*D*). As expected, the WT diploid XL280 α/**a** strain sporulated robustly on V8 medium (*SI Appendix*, Fig. S8*E*).In contrast, the diploid *msd1*Δα/*msd1*Δ**a** mutant failed to form spores even after prolonged incubation (>1 mo) (*SI Appendix*, Fig. S8*E*). Consistently, the diploid *msd1*Δα/*msd1*Δ**a** mutant exhibited a significant lower BMS level compared to the XL280 α/**a** strain (*SI Appendix*, Fig. S8*F*). Furthermore, transcript levels of the meiosis-specific genes *DMC1* and *REC8* were similarly dramatically reduced in the diploid *msd1*Δα/*msd1*Δ**a** mutant compared to that in the XL280 α/**a** strain on V8 media (*SI Appendix*, Fig. S8*G*). Collectively, these results indicate that Msd1 is indispensable for initiating meiosis and sporogenesis regardless of the ploidy state.

### 1.4. Msd1 Overexpression Is Sufficient to Activate Meiosis, and This Function Is Conserved Across the *Cryptococcus* Species Complex.

**Fig. 3.**
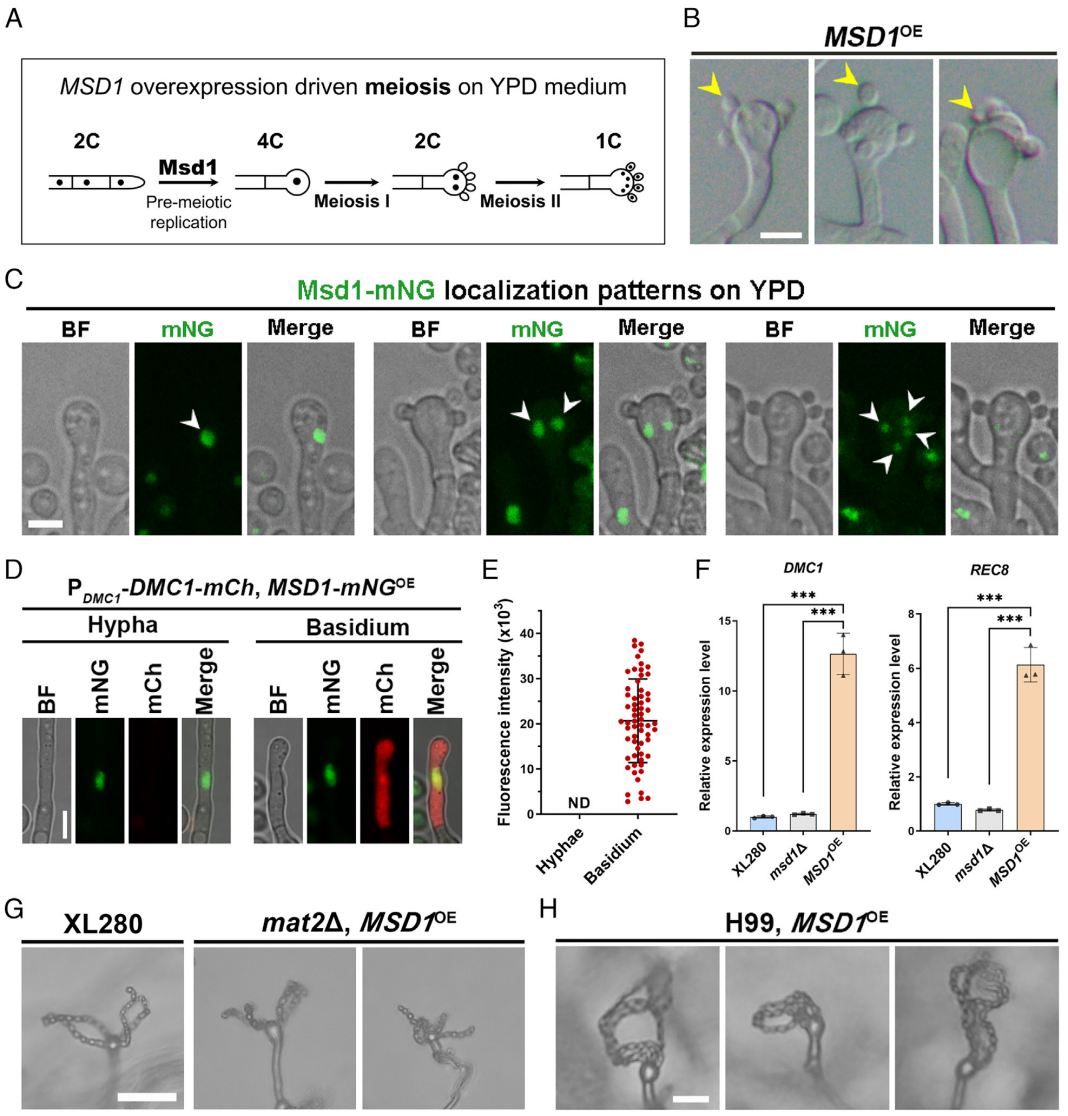
Overexpression of *MSD1* is sufficient to drive meiosis and sporulation under mating-repressing condition and across various genetic backgrounds. (*A*) Schematic diagram illustrating that *MSD1* overexpression drives meiosis and sporogenesis under mating repressing condition. (*B*) Morphology of the hyphal tips from the *MSD1*^OE^ strain cultured on YPD medium for 2 d. The yellow arrows indicate the basidiospores. (Scale bar, 10 μm.) (*C*) Subcellular localization pattern of Msd1-mNG on YPD medium driven by the *GPD1* promoter. The white arrows indicate the Msd1-mNG signal within the basidium. BF and mNG indicate Brightfield and mNeonGreen, respectively. (Scale bar, 5 μm.) (*D*) Overexpression of *MSD1* drives the spatiotemporal expression of *DMC1-mCherry* within the basidium, but not in the subapical hyphal compartment. mCh and mNG indicate mCherry and mNeonGreen, respectively. (Scale bar, 5 μm.) (*E*) Quantification of the Dmc1-mCherry signal from the subapical hyphal compartment and the basidium of the Msd1-mNG overexpression strain as in panel D (n = 60). ND, not detected. (*F*) RT-qPCR assay of the relative transcript level of *DMC1* and *REC8* in the WT, *msd1*Δ, and *MSD1*^OE^ strains on YPD medium. Error bars represent mean ± SD from three biological replicates. Statistical significance assay was performed by two-tailed Student’s *t* test (***P < 0.001) (*G*) Overexpression of *MSD1* in the *mat2*Δ background restored sporulation after incubated on V8 medium for 2 wk. (Scale bar, 20 μm.) (*H*) Canonical spore chains were observed in the recombinant H99 strain overexpressing *MSD1* under mating inducing condition. (Scale bar, 10 μm.)

The above results demonstrate that Msd1 is necessary for activating meiosis during sexual reproduction. If Msd1 functions as a master regulator of meiosis, its overexpression should be sufficient to drive meiosis and sporogenesis independently of the mating pathway or external mating cues (Fig. 3*A*). Indeed, we found that *MSD1* overexpression effectively induced yeast-hypha morphogenesis on the mating-suppressive YPD medium (*SI Appendix*, Fig. S3*D*). Remarkably, enlarged basidium heads were observed from the *MSD1*^OE^ strain after 2 d of incubation on YPD medium (Fig. 3*B*). Moreover, small spore-like cells emerged from these enlarged hyphal tips (yellow arrows in Fig. 3*B*). This result suggests that overexpression of *MSD1* is able to drive basidial differentiation and sporogenesis events under mating-repressing condition.

During meiosis, chromosomes undergo a single round of replication followed by two successive rounds of cell division, known as meiosis I and meiosis II. Therefore, if meiosis occurs, we expect to observe dynamic changes in nuclear number, including the emergence of two and four nuclei (Fig. 3*A*). Indeed, after 2 d of incubation on YPD medium, we observed the presence of a single nucleus, two nuclei, or four nuclei within a single basidium in the *MSD1*^OE^ strain based on the nuclear Msd1-mNG fluorescence signal (white arrows in Fig. 3*C*), consistent with the occurrence of meiosis I and meiosis II. Furthermore, small spores emerged only from basidial structures that contained two or four nuclei, not from those with only a single nucleus (Fig. 3*C*). Consistently, Dmc1, the well-established molecular marker of meiosis, was highly expressed within the basidium, but not in the subapical hyphal compartment of the *MSD1-mNG*^OE^ strain cultured on the YPD medium (Fig. 3 *D* and *E*). RT-qPCR assay also revealed a significant up-regulation of *DMC1* and *REC8* genes on YPD medium when *MSD1* was overexpressed (Fig. 3*F*). Collectively, these findings indicated that forced expression of Msd1 is sufficient to drive meiosis and subsequent sporogenesis even under a mating-suppressive condition.

Given that Msd1 overexpression is capable of driving meiosis under mating-repressing condition, we asked whether it could bypass the Mat2 controlled pheromone-pathway to trigger meiosis and sporulation. In the *mat2*Δ mutant, *MSD1* overexpression partially restored filamentation (*SI Appendix*, Fig. S9*A*) and the expression of morphogenesis genes *ZNF2*, *CFL1*, and *PUM1* on V8 medium (*SI Appendix*, Fig. S9*B*). Remarkably, it fully rescued the sporulation defect (Fig. 3*G*) and drastically induced the expression of meiotic genes *DMC1* and *REC8* (*SI Appendix*, Fig. S9*C*).By contrast, none of the pheromone pathway genes were activated (*SI Appendix*, Fig. S9*D*). These results indicate that Msd1 overexpression bypasses the requirement of the pheromone pathway to activate meiosis and sporogenesis, consistent with its ability to drive meiosis and sporulation under mating-suppressive condition. To determine if Msd1’s function is conserved across *Cryptococcus* species, we decided to examine its role in the *C. neoformans* serotype A reference strain H99 and also in a sibling species *C. gattii* reference strain R265. To this end, we first generated an *MSD1* overexpression strain in the H99 background. When cultured on V8 medium alone, the *MSD1*^OE^ (H99) strain underwent robust hyphal growth (*SI Appendix*, Fig. S10), and these hyphae subsequently differentiated into basidial heads and generated spore chains after 1 mo of incubation (Fig. 3*H*). In contrast, the WT H99 strain was completely nonfilamentous due to its inability to undergo unisexual reproduction on V8 medium. When cultured on the mating-suppressing YPD medium, hyphae generated from the *MSD1*^OE^ (H99) strain differentiated into enlarged basidium heads but failed to sporulate even after extended incubation (*SI Appendix*, Fig. S11*A*). Consistent with this finding on YPD medium, basidium heads were also formed in the *MSD1*^OE^ (H99) strain under mating-inducing condition when Mat2 is absent. Nonetheless, no spores were generated after extending the incubation on V8 media to 1 mo (*SI Appendix*, Fig. S11*B*). These results indicated that the role of Msd1 in activating meiosis and basidium maturation was conserved. However, the regulatory circuit in driving postmeiotic sporogenesis may get rewired in H99. When we overexpressed *MSD1* ortholog, *CgMSD1*, in the non-self-filamentous *C. gattii* R265 background, we observed efficient yeast-hypha morphogenesis on V8 or YPD medium (*SI Appendix*, Fig. S12*A*). After 1 wk of incubation on V8 medium, spores emerged from the hyphal apex of the *CgMSD1*^OE^ (R265) strain on mating-inducing V8 medium (*SI Appendix*, Fig. S12*B*). Remarkably, the *CgMSD1*^OE^ (R265) strain also generated spores when cultured on mating-repressing YPD medium (*SI Appendix*, Fig. S12*B*). These data indicated that overexpression of *CgMSD1* is sufficient to drive meiosis and sporogenesis both under mating-inducing and -repressing conditions in *C. gattii*, similar to what we observed in the serotype D XL280 strain. Thus, Msd1 shows a conserved role as a master regulator of meiosis initiation across the pathogenic *Cryptococcus* species complex.

### 1.5. Msd1 Is Responsible for the Activation of Conserved Genes for Meiosis and Sporulation.

**Fig. 4.**
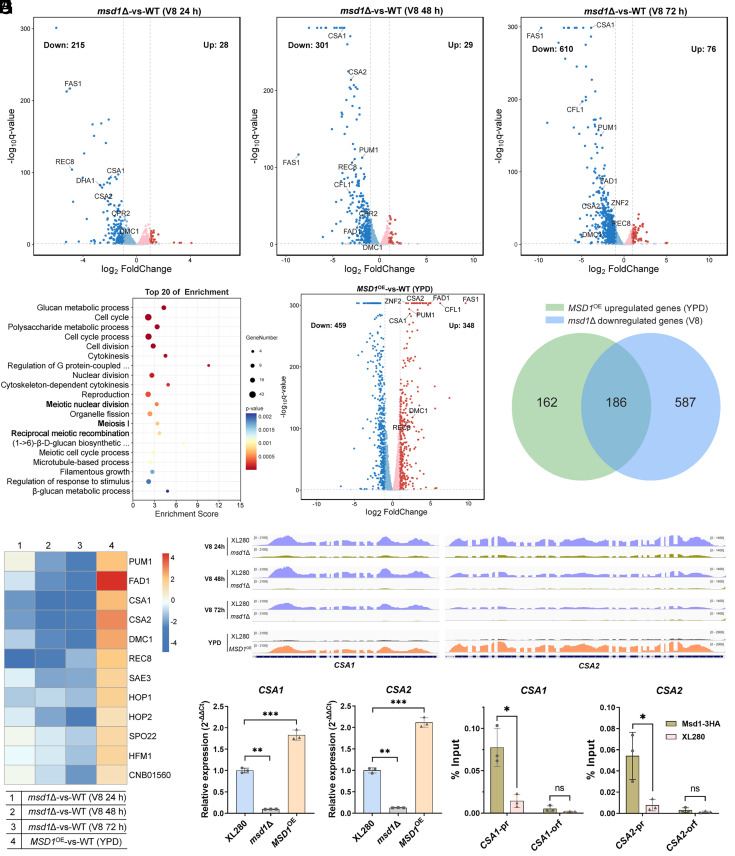
Msd1 activates a variety of genes involved in meiosis and sporulation. (*A*) Volcano plots exhibited the differentially expressed genes (DEGs) in the *msd1*Δ mutant compared to WT strain cultured on V8 media for 24 h, 48 h, and 72 h, respectively. (*B*) The top 20 enriched Gene Ontology (GO) terms (Biological Processes) of the Msd1 regulon on V8 medium. (*C*) Volcano plots illustrating the DEGs in the *MSD1* overexpression strain compared to WT under mating repressing condition (YPD). (*D*) Venn diagram analysis between the down-regulated genes of the *msd1*Δ mutant derived from V8 medium and the up-regulated gene of the *MSD1* overexpression strain on YPD medium. (*E*) Heatmap of the sporulation and meiosis genes in the indicated groups. Color bar indicated log_2_(fold change) value. (*F*) Reads coverage of *CSA1* and *CSA2* gene loci in the indicated strains and culture conditions. (*G*) RT-qPCR assay of *CSA1* and *CSA2* genes in XL280, *msd1*Δ mutant, and *MSD1* overexpression stains cultured on V8 medium for 24 h. Error bars represent the mean ± SD from three biological replicates. Statistical significance assay was performed by two-tailed Student’s *t* test (**P < 0.01; ***P <0.001). (*H*) ChIP-qPCR assay demonstrated the binding of Msd1 to *CSA1* and *CSA2* promoter in vivo. *P < 0.05; ns, not significant; two-tailed Student’s *t* test.

To further elucidate the role of this transcription factor in regulating meiosis and sporogenesis, we performed an RNA-seq assay with the *msd1*Δ mutant and the wildtype XL280 cultured on V8 media for 24 h, 48 h, and 72 h, respectively, which covered the early, the middle, and the late stages of sexual development. As shown in Fig. 4*A*, a total of 243, 330, and 686 DEGs were identified between the mutant and the WT at these three time points, respectively (threshold log_2_|fold-change| > 1.0 and q value < 0.01, Dataset S1). Among these DEGs, the majority of them were down-regulated in *msd1*Δ mutant at each time point [215 (88.5%), 301 (91.2%), and 610 (88.9%), respectively], indicating that Msd1 predominantly functions as a transcriptional activator during sexual development. A total of 773 genes were significantly downregulated in the *msd1*Δ mutant at least at one time point. We thus defined these genes as the Msd1 regulon under the V8 culture conditions. A GO analysis revealed that the Msd1 regulon is highly enriched in cell cycle/GO:0007049, cell division/GO:0051301, cytokinesis/GO:0000910, and meiotic progression, including meiotic nuclear division/GO:0140013, meiosis I/GO:0007127, and reciprocal meiotic recombination/GO:0007131 (Fig. 4*B*). The transcriptome result further supports the critical role of Msd1 in activating meiosis.

As *MSD1* overexpression is sufficient to drive sexual development under mating-suppressing conditions, we hypothesized that Msd1’s core targets would be downregulated in the *msd1*Δ mutant under mating-inducing condition (on V8) and up-regulated in the *MSD1*^OE^ strain under mating-repressing condition (on YPD). To identify these genes, we performed an additional RNA-Seq experiment with *MSD1*^OE^ strain and the wildtype XL280 cultured on YPD medium. 348 and 459 genes were significantly up- or down-regulated in the *MSD1*^OE^ strain compared to XL280, respectively (Fig. 4*C*). A Venn diagram analysis revealed that 186 genes were shared between the Msd1 regulon on V8 medium (773 down-regulated genes in *msd1*Δ) and on YPD medium (348 up-regulated genes in *MSD1*^OE^) (Fig. 4*D*). We defined this set of genes as the Msd1 core regulon (Dataset S2). As expected, this core regulon encompasses a variety of meiotic genes, including the well-characterized meiosis-specific genes *DMC1* and *REC8*, as well as six additional annotated meiotic genes: *SAE3* (*CNE00220*), *HOP1* (*CNK00900*), *HOP2* (CNK01660), *SPO22* (*CNI02810*), *HFM1* (*CNI00970*), and *CNB01560* (Fig. 4*E*). This core regulon also included a well-characterized secretory protein, Fad1 (Fig. 4*E*), which was predominantly expressed in basidium and participated in postmeiotic sporulation (35). Moreover, putative Msd1 binding motifs (HGATAR) were found at the promoter region of all these meiosis and sporulation genes examined (*SI Appendix*, Fig. S13). Thus, Msd1 regulates the core meiotic genes as well as postmeiotic genes.

To determine if Msd1 has downstream sentinel TFs in initiating meiosis, we identified 11 putative TF-encoding genes within the core regulon (*SI Appendix*, Fig. S14*A*). We constructed individual knockout strains for these TF genes and evaluated their sporulation phenotype on V8 medium. None of these TF mutants were found to be important for hyphal development or sporulation (*SI Appendix*, Fig. S14 *B* and *C*). As meiosis is the prerequisite for sporulation, these findings suggested that Msd1 may act as the terminal master transcription factor in activating meiosis.

Since none of the Msd1-regulated TFs were found to be required for sporulation, we directed our attention to downstream RNA-binding protein(s) regulated by Msd1, as it has been reported that RNA-binding proteins are involved in meiotic entry across from fission yeast to mammals (11, 39, 40). Notably, two RNA-binding protein encoding genes, *CSA1* (*CNJ00760*) and *CSA2* (*CNB02060*), critical for meiosis and basidial maturation (35), were markedly down-regulated in the *msd1*Δ mutant at all three time points on V8 medium (Fig. 4 *E* and *F*). Conversely, overexpression of *MSD1* dramatically enhanced the expression of these two genes under mating-repressing condition (Fig. 4*F*). The transcriptional regulation of these two genes by Msd1 was further validated through RT-qPCR assay (Fig. 4*G*). To determine if Msd1 directly activates *CSA1* and *CSA2*, we performed a ChIP-qPCR assay with the Msd1-3xHA overexpression strain. Indeed, Msd1 enrichment signal was detected in the promoter region, but not the ORF region of *CSA1* and *CSA2*, suggesting a direct regulatory role of Msd1 in activating their expression (Fig. 4*H*).

### 1.6. Msd1 Targets Two RNA-Binding Proteins, Csa1 and Csa2, in Controlling Basidial Differentiation and Meiosis Initiation.

**Fig. 5.**
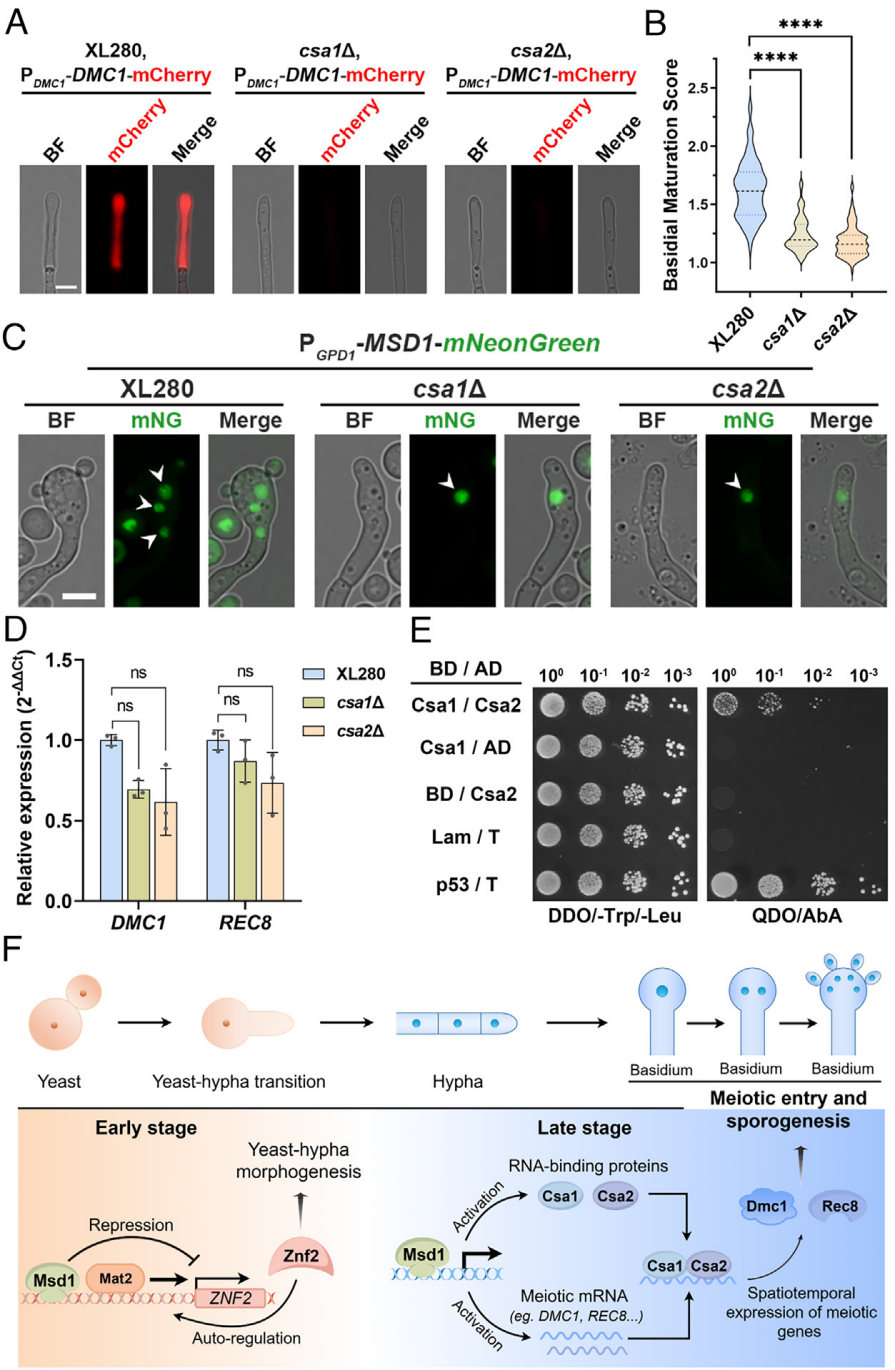
Msd1 initiates meiosis by targeting two conserved RNA-binding proteins, Csa1 and Csa2. (*A*) Deletion of *CSA1* and *CSA2* compromised the spatiotemporal expression of the meiotic recombinase gene *DMC1*. (Scale bar, 10 μm.) (*B*) Deletion of *CSA1* and *CSA2* blocked basidial maturation during sexual development. Statistical significance assay was determined by two-tailed Student’s *t* test (****P < 0.0001). (*C*) Overexpression of *MSD1* in the *csa1*Δ and *csa2*Δ mutant background failed to activate meiosis and sporogenesis under mating repressing condition. (Scale bar, 5 μm.) (*D*) RT-qPCR assay of the relative transcript level of *DMC1* and *REC8* in the WT, *csa1*Δ, and *csa2*Δ strains on V8 medium. Error bars represent mean ± SD from three biological replicates. ns, not significant, two-tailed Student’s *t* test. (*E*) Csa1 interacts with Csa2 in yeast-two hybrid assay. The QDO medium (SD/-Trp/-Leu/-His/-Ade) supplemented with 150 ng/mL AbA was used for detecting the interaction of the indicated protein pairs. Lam/T and p53/T were set up as negative and positive controls, respectively. (*F*) Schematic model of Msd1 in activating meiotic entry in *C. neoformans*. During the early sexual development stage, yeast-hypha morphogenesis is driven by Znf2, which is predominantly activated by Mat2 and modestly repressed by Msd1. During the late stage of sexual reproduction, Msd1 plays a dominant role in controlling meiotic entry by activating two interconnected pathways: transcriptional activation of meiotic genes *DMC1* and *REC8*, and expression of two RNA-binding proteins, Csa1 and Csa2. Csa1 and Csa2 work coordinately to facilitate the spatiotemporal expression of the core meiotic protein within the basidium, thus enabling the meiotic entry and sporogenesis. This diagram was drawn using the Figdraw online program.

Both *CSA1* and *CSA2*-encoded proteins contain two RNA-Recognition Motifs (RRMs) (*SI Appendix*, Fig. S15*A*), the most common RNA-binding domain in eukaryotes. Phylogenetic analysis revealed that Csa1 and Csa2 homologs are widely distributed in ascomycetes and basidiomycetes across the fungal kingdom (*SI Appendix*, Fig. S15*B*). Similar to the *msd1*Δ mutant, the Dmc1-mCherry signal was barely detectable in the *csa1*Δ and *csa2*Δ mutants during sexual development (Fig. 5*A*). Likewise, their basidium differentiation was defective, as evidenced by dramatically decreased BMS levels (Fig. 5*B*). Comprehensive phenotypic assays revealed that the *csa1*Δ and *csa2*Δ mutants behaved similarly to the WT strain under various conditions tested (*SI Appendix*, Fig. S16), indicating that Csa1 and Csa2 likely function specifically in activating meiosis in *C. neoformans*. To further investigate the involvement of Csa1 and Csa2 in activating meiosis, we generated *MSD1*-*mNG* overexpression strains in the *csa1*Δ and *csa2*Δ mutant background. In the WT background, meiotic nuclear division was observed upon *MSD1*-mNG overexpression (Fig. 5*C*). In contrast, overexpression of *MSD1*-mNG in the *csa1*Δ and *csa2*Δ mutants failed to drive meiotic nuclear division, as only a single nucleus was observed within the hyphal tips of these strains (Fig. 5*C*). These results indicate that Csa1 and Csa2 are two critical downstream targets of Msd1 in activating meiosis. Intriguingly, RT-qPCR assay showed that disruption of *CSA1* and *CSA2* hardly affects the transcription level of *DMC1* and *REC8* genes (Fig. 5*D*), or the splicing of their mRNAs (*SI Appendix*, Fig. S17). These results indicated that Csa1 and Csa2 may regulate the expression of these two meiotic genes posttranscriptionally, such as facilitating their translation. Given that Csa1 and Csa2 behaved similarly, we speculate that these two proteins may function in a complex to regulate meiosis. Indeed, Csa1 and Csa2 showed positive interaction based on a yeast two-hybrid assay (Fig. 5*E*), supporting a direct physical interaction between these two RNA-binding proteins. Taken together, these results indicate that Msd1 controls meiosis initiation not only through activating the core conserved meiotic genes such as *DMC1* and *REC8* but also through two downstream RNA-binding proteins, which may function to ensure translation of these core meiosis transcripts. Such dual effects enable Msd1 to initiate the highly conserved meiotic process in a manner unique to this basidiomycete (Fig. 5*F*).

## 2. Discussion

Meiosis is a crucial process for gametogenesis. Compared to the faithful passage of nuclear DNA from mitosis, meiosis drives the generation of genetic and karyotypic diversity through de novo mutations (41, 42), chromosome crossover (43), and meiotic recombination (21, 22), all of which facilitate adaptation and species evolution (24). In *C. neoformans*, extensive meiotic recombination occurs during both unisexual and bisexual reproduction, contributing to the emergence of clinically highly virulent and drug-resistant progenies (21, 22, 25, 44). The final products of sexual reproduction, basidiospores, are infectious propagules due to their small size and stress-resistance (26, 27, 45). Despite extensive investigations into its sexual cycle in the past decades (20, 31, 32, 36, 46–48), the master regulator governing meiotic entry remained unknown. Here, we identified a novel transcription factor Msd1 that is responsible for the activation of meiotic entry in this pathogenic fungus and demonstrated that Msd1 fits the definition of “master regulator” (49): 1) *MSD1* is expressed at the inception of cryptococcal sexual development and also throughout the entire sexual development process; 2) Its deletion impairs hyphal development and basidial maturation, and completely abolishes meiosis; 3) Ectopic overexpression of *MSD1* is sufficient to drive meiosis even under mating repressing condition, whereas the cell fate of the wild type is restricted to vegetative yeast growth; 4) Msd1’s function in initiating meiosis is conserved in the pathogenic *Cryptococcus* species complex.

Studies of meiosis initiation in the model ascomycete yeasts and mammals illustrate the remarkable plasticity in regulating this fundamental process. For instance, homologs of the *S. cerevisiae* master transcription factor Ime1 for meiosis entry are not found in any other distantly related fungal species. Similarly, Msd1 homologs are only distributed in the Tremellales order within the Basidiomycota phylum. In *S. pombe*, meiosis initiation is controlled at the posttranscriptional level by an RNA-binding protein Mei2 (11, 14), highlighting the crucial role of posttranscriptional regulation in the control of meiotic entry. In mammals, two transcription factors, Stra8 and MEIOSIN, synergistically control the meiotic initiation in germ cells in response to retinoic acid (50–52). Posttranscriptional regulation also plays a crucial role in mitotic-to-meiotic transition during spermatogenesis. Specifically, the RNA helicase YTHDC2, in cooperation with its interacting partners MEIOC and an RNA-binding protein RBM46, selectively facilitates the degradation of mitotic transcripts, which consequently allows the establishment of a meiotic transcriptome during mammalian spermatogenesis (39, 40). Regulation at both the transcriptional and the posttranscriptional level might have enabled a much tighter control of meiotic entry, which is likely more critical for a complex multicellular organism. In the basidiomycete *C. neoformans*, we demonstrate that Msd1 directly controls the transcription of the core conserved meiotic genes, such as *DMC1* and *REC8*. Meanwhile, Msd1 also directly activates the expression of two RNA-binding proteins Csa1 and Csa2, which ensure the spatiotemporal expression of the core meiotic genes in the basidial heads. By controlling the expression of its regulon, Msd1 directly provides at least two layers of reinforcement to push through the meiotic program in a specific manner unique to this basidiomycete (Fig. 5*F*). It is worth noting that, in addition to its predominant role in activating meiotic entry, Msd1 also contributes to hyphal morphogenesis by regulating *ZNF2* expression, which is also regulated by Mat2. However, the regulatory role of Msd1 in these two processes cannot be separated completely and its relationship with Znf2 is rather complex. The regulatory complexity is likely required to coordinate multiple cellular differentiation events during sexual reproduction and eventually achieve the commitment to meiosis and sporogenesis.

*C. neoformans* typically exists in the haploid state in vitro but can undergo polyploidization during infection (53, 54), a feature implicated in cryptococcal dormancy and persistence in the host lungs. We demonstrated previously that activation of the highly conserved meiotic genes, such as *DMC1* and *REC8*, contributes to cryptococcal ploidy reduction during infection, thereby facilitating the generation of haploid progeny that are antigenotoxic and adapted to the host-relevant stressful conditions (28). In mammalian cancer cells, meiosis genes such as *DMC1* similarly participate in the ploidy reduction process that generates diploid daughter cells recalcitrant to antitumor treatment (55–57). Thus, reversible ploidy changes mediated by meiosis or a meiosis-like mechanism may be widely conserved in eukaryotic adaptation to complex stress. Whether Msd1 activates meiosis genes during infection and thus contributes to reactivation of a dormant cryptococcal infection would be an interesting topic to investigate in the future.

## 3. Materials and Methods

### 3.1. Strains, Medium, and Growth Conditions.

*Cryptococcus* strains were routinely cultured at 30 °C on YPD medium (1% yeast extract, 2% peptone, 2% glucose, and plus 2% agar for solid plate) supplemented with selective drugs (100 μg/mL nourseothricin, 100 μg/mL G418, or 200 μg/mL hygromycin), when necessary. Unisexual and bisexual mating experiments were performed on V8 juice agar medium [0.5 g/L KH_2_PO_4_, 5% V8 juice (v/v), 4% Bacto agar, adjusting pH to 7.0 for serotype D strains and 5.0 for serotype A strains] at 25 °C in the dark for the indicated time period. The *S. cerevisiae* strains Y187 and Y2H Gold were utilized for yeast one-hybrid screening and yeast two-hybrid assay, respectively. *S. cerevisiae* yeast cells were routinely cultured at 30 °C on YPDA medium (YPD medium supplemented with 60 μg/mL adenine). Synthetic Complete (SC) medium lacking tryptophan (Trp) and leucine (Leu), supplemented with 100 ng/mL of AbA, was employed for screening of positive clones.

Strains used in this study are listed in *SI Appendix*, Table S2. Details about strains and plasmids construction are included in the *SI Appendix*.

### 3.2. Cryptococcal cDNA Library Construction and Y1H Screen.

For cryptococcal cDNA library construction, XL280 cells cultured under various growth conditions, including YPD, V8, V8 + 500 μM Cu^2+^, and YP-GlcN, were collected individually for total RNA extraction. After DNase treatment, these RNA samples were combined together, and the mixture was sent to Bio S and T Inc. (Quebec, Canada) for cDNA library construction via cloning into the pGADT7 plasmid.

For Y1H screening, 10 µg of *C. neoformans* cDNA library plasmid was transformed into the yeast bait strain using a commercial yeast transformation kit (CAT#630439, Clontech, TAKARA). The potential positive clones were then screened on a synthetic SD medium plate lacking Leu and Trp but containing 100 ng/mL AbA (DDO/-Trp/-Leu/AbA). The clones were cultured on the selective plate for 3 d at 30 °C. Then, colony PCR was performed using the universal primers T7/3′-AD of the clones appeared on the selective plate to obtain the inserted cDNA fragments. Subsequently, the PCR products were sent for Sanger sequencing and the sequences were used to identify the inserted genes through BLASTN alignment on the FungiDB website (https://fungidb.org/fungidb/app).

### 3.3. Basidial Maturation and Sporulation Assay.

Basidium maturation assay was performed as previously described (35). Briefly, the hypha cells from the edge of the mating colony that had been incubated on V8 medium for 2 wk were collected and suspended in 100 μL 1 × PBS buffer. The cell suspensions were then vortexed and 10 μL for each strain were dropped onto a glass slide and observed under an optical microscope with a AxioCam ERc 5 s camera (AXIO lab.A1, Zeiss). The diameter of the basidium and its connected subapical hypha compartment were measured with the software Zen 2011 (Carl Zeiss). The BMS value was determined by calculating the diameter ratio between the basidium and its connected hypha as described previously (35). One hundred hyphal tips from each strain were randomly selected for the BMS measurement.

For the sporulation assay, the unisexual or bisexual mating colonies were incubated on V8 medium at 25 °C in the dark for 2 to 3 wk. The morphology of the hyphal tips with or without spore chains were photographed under an optical microscope (BX43, Olympus).

### 3.4. Blastospore Dissection and Ploidy Assay.

Blastospores were dissected from the unisexual mating colonies cultured on V8 medium for 2 wk using a dissection microscope (SporePlay^+^, Singer Instruments). Ploidy of cells amplified from the dissected blastospores were determined by PI staining and flow cytometry analysis as previously described (28). Flow cytometry assay was performed with 10,000 cells for each sample and analyzed on the FL1 channel on a CytoFLEX S flow cytometer (Beckman Coulter) at Jiangxi Institute of Respiratory Diseases. FlowJo (v10.0) software was used for data analysis.

### 3.5. Microscopic Fluorescence Observation.

To monitor the expression and subcellular localization dynamics of mNG tagged Znf2 and Msd1 during unisexual reproduction, the *mNG*-*ZNF2* and *MSD1-mNG* knock-in strains were spotted onto V8 agar plate and incubated at 25 °C in the dark. The cells of each strain were scraped from the colony edge for observation at 24 h, day 3, day 5, and day 10, covering stage I to stage V during unisexual reproduction. Cells were examined under a Thunder fluorescence microscope (DMi8, Leica) with Las X software. Fluorescent intensity of at least 30 nuclei per developmental stage for each strain was quantified using LAX software.

### 3.6. Chromatin Immunoprecipitation (ChIP).

ChIP assay was performed as previously described (34). Detailed technical processes about ChIP assay are included in the *SI Appendix*. All primers used for ChIP-qPCR assay are listed in *SI Appendix*, Table S3. The relative enrichment of DNA fragments was determined as a percentage of input DNA (Input%) based on RT-qPCR analysis, as previously described (34).

### 3.7. Recombinant Protein Purification and EMSA.

For the purification of Msd1 DNA-binding domain (Msd1^DBD^, amino acids 234 to 306) in *E. coli*, the DNA fragment encoding Msd1^DBD^ was amplified from XL280 cDNA and cloned into the pET-GST-6His expression vector via EcoRI/SalI restriction digestion followed by seamless cloning, resulting in the recombinant plasmid pET-GST-Msd1^DBD^-6His. This final plasmid and the original plasmid pET-GST-6His were individually transformed into *E*. *coli* Rosetta (DE3) cells for recombinant protein expression. The *E. coli* strains were cultured in LB medium supplemented with 40 μg/mL kanamycin at 37 °C until the cell density reached an OD600 of 0.5 to 0.6. Recombinant protein expression was induced by adding isopropyl β-D-thiogalactoside (IPTG) to a final concentration of 0.5 M, followed by incubation at 20 °C for 16 h. Protein purification was performed by using a His-tag Protein Purification Kit (P2226, Beyotime, Shanghai, China) by following the manufacturer’s instructions. SDS-PAGE was employed to evaluate the molecular weight and purity of the purified recombinant protein, and protein concentration was determined by using a bicinchoninic acid (BCA) protein assay kit (A65453, Thermo Fisher). EMSA was performed by using a commercial Chemiluminescent EMSA Kit (GS009, Beyotime, Shanghai, China) according to the manufacturer’s protocol.

### 3.8. RNA Purification and RT-qPCR Assay.

For RNA extraction, *Cryptococcus* strains were cultured in YPD liquid media, collected by centrifugation, and washed with sterile water. The cell suspension was spotted onto YPD or V8 plate and incubated at 25 °C in the darkness for 24 h. The cells were then harvested, washed twice with cold PBS buffer and stored at−80 °C. Total RNA was extracted with a commercial Ultrapure RNA Kit (CW0581M, CWBIO) according to the manufacturer’s instructions. After DNase I treatment, reverse transcription was performed with 1 μg RNA from each strain using the HiScript III RT SuperMix kit (R323-01, Vazyme) according to the manufacturer’s protocol. The RT-qPCR was performed using the ChamQ Universal SYBR qPCR Master Mix (Q711, Vazyme) in an ABI RT-qPCR system (ABI QuantStudio Dx). Three biological replicates were included for each sample, and the *TEF1* gene was used as an endogenous control for internal normalization to determine the relative transcript levels (ΔΔCt method) of the examined genes as previously described (33). All the primers used for RT-qPCR are listed in *SI Appendix*, Table S3.

### 3.9. RNA-Seq.

For RNA-Seq assay, the *msd1*Δ mutant and XL280 strains cultured on V8 medium for 24, 48, and 72 h were harvested, respectively. In addition, the *MSD1* overexpression strain and XL280 cultured on YPD were also collected. Three biological replicates were prepared for each strain under each condition. Total RNA extraction was performed by using the Ultrapure RNA Kit (CW0581S, CWBIO, China) according to the manufacturer’s protocol. Subsequently, the total RNA was treated with the TURBO DNA-free™ Kit (AM1907, Thermo Fisher) to digest the genomic DNA prior to library construction. The Agilent 2100 Bioanalyzer (Agilent Technologies, Santa Clara, CA) was used for RNA quality and integrity evaluation. Libraries were constructed using the VAHTS Universal V6 RNA-seq Library Prep Kit (NR604, Vazyme, Nanjing, China) following the manufacturer’s instructions. Transcriptome sequencing and data analysis were performed by OE Biotech Co., Ltd. (Shanghai, China) with the Illumina Novaseq 6000 platform. The online bioinformatics tools (https://cloud.oebiotech.cn/task/) of the OE Biotech were used for volcano plot, Venn diagram, heatmap, and GO enrichment analysis and visualization.

## Supplementary Material

Appendix 01 (PDF)

Dataset S01 (XLSX)

Dataset S02 (XLSX)

## Data Availability

RNA-Seq data have been deposited in NCBI Gene Expression Omnibus (GEO) (GSE308021) (58). All study data are included in the article and/or supporting information.
