# Supplementary material for: Identification of a master regulator Msd1 that governs meiotic entry in a global basidiomycete pathogen
Source: Proc Natl Acad Sci U S A. 2026 Jun 24;123(26):e2536339123. doi: 10.1073/pnas.2536339123 (PMC13321354; doi:10.1073/pnas.2536339123)
Supplement: Supplementary file 1 — Appendix 01 (PDF) [file pnas.2536339123.sapp.pdf]

PNAS

Supporting Information for

Discovery of a master regulator Msd1 that governs meiotic entry in a global basidiomycete pathogen

Fanglin Zheng[1, 2, 3, 7] *, Yanli Cao[4], Huiting Chen[1], Lili Yan[1, 2, 3], Feng Lv[1], Yuan Huang[5], Man Chen[1], Lin Su[5], Zhuozhuo Liu[1], Ye Huang[1, 2, 3], Tuyetnhu Pham[6], Xinping Xu[1, 2, 3, *], Xiaorong Lin[6,7, *]

[1]Jiangxi Provincial Key Laboratory of Respiratory Diseases, Jiangxi Institute of Respiratory Diseases, The Department of Respiratory and Critical Care Medicine, The First Affiliated Hospital, Jiangxi Medical College, Nanchang University, Nanchang, Jiangxi 330006, China.[2]Jiangxi Clinical Research Center for Respiratory Diseases, Nanchang, Jiangxi 330006, China.
[3]Jiangxi Hospital of China-Japan Friendship Hospital, Nanchang, Jiangxi 330006, China.
[4]Department of immunology, School of Medicine, Jiangxi Medical College, Nanchang University, Nanchang, Jiangxi 330006, China.
[5]Department of Geriatric Medicine, The First Affiliated Hospital, Jiangxi Medical College, Nanchang University, Nanchang, Jiangxi 330006, China.
[6]Department of Plant Biology, University of Georgia, Athens, GA 30602, USA.
[7]Department of Microbiology, University of Georgia, Athens, GA 30602, USA.

*Correspondence: fanglin.zheng@ncu.edu.cn (F.Z.), xinpingxu@ncu.edu.cn (X.X.), xiaorong.lin@uga.edu (X.L.).

This PDF file includes:

Figures S1 to S17
Tables S1 to S3
Method (Strains, plasmids construction and ChIP)
SI References

Other supporting materials for this manuscript include the following:

Dataset S1
Dataset S2

Figures

Fig. S1

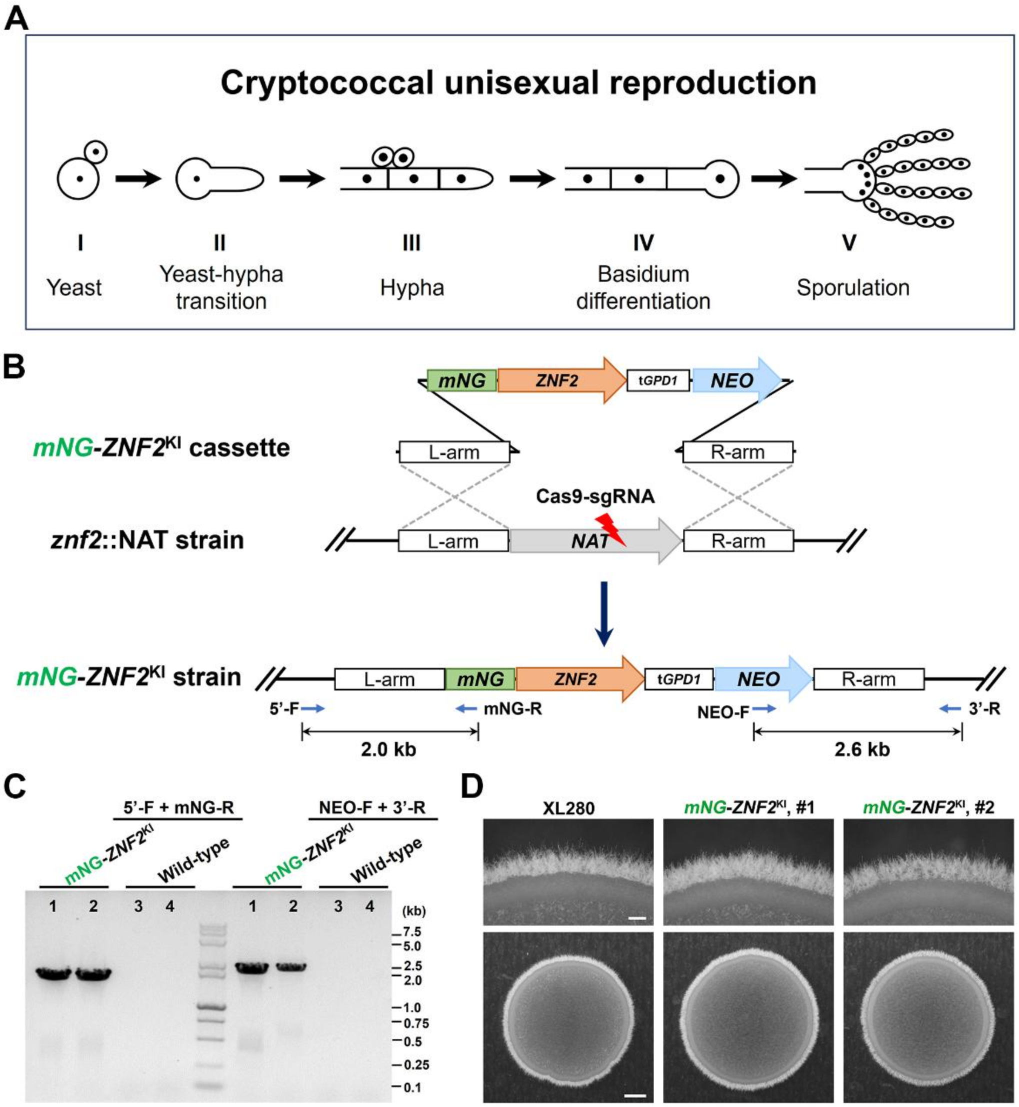

Fig. S1 Construction of the *mNeonGreen-ZNF2* knock-in (*mNG-ZNF2*^{KI}) strain. (**A**) Schematic representation of the five cellular development stages during cryptococcal unisexual development. (**B**) Schematic diagram illustrating the procedure for generating the *mNG-ZNF2*^{KI} strain. The knock-in cassette harbors an N-terminal mNG tagged *ZNF2* under control of its native promoter (L-arm), a G418 selective marker (*NEO*) and *ZNF2* 3'-flanking sequence (R-arm). This DNA construct was transformed into a *znf2*Δ mutant (*znf2*::NAT) and integrated at the *ZNF2* locus via the CRISPR-Cas9 system mediated double stranded break and homology-based repair. (**C**) Diagnostic PCR confirmation of the *mNeonGreen-ZNF2* knock-in strain. The primers used in PCR and the product sizes are indicated in panel B. Two independent positive clones were shown here. (**D**) Filamentation assay of the *mNG-ZNF2*^{KI} strains during unisexual reproduction. The images were captured after three days of incubation on V8 medium at 25℃ in the dark. Scale bar, upper panel 0.2 mm, bottom panel 1 mm.

Fig. S2

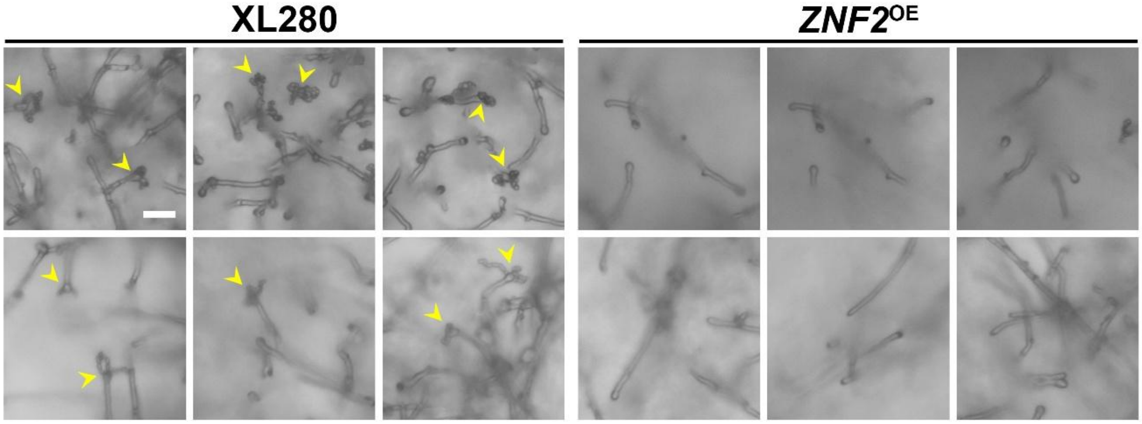

Fig. S2 Overexpression of *ZNF2* suppresses sporulation. The wild-type strain XL280 and *ZNF2* overexpression strain (P_{GPD1}-*ZNF2*) were cultured on V8 medium, and images were captured after two weeks of incubation. Yellow arrows indicate basidium heads with spores or spore chains, which were abundant in XL280 but rarely detected in the *ZNF2*^{OE} strain. Scale bar, 20 μm.

Fig. S3

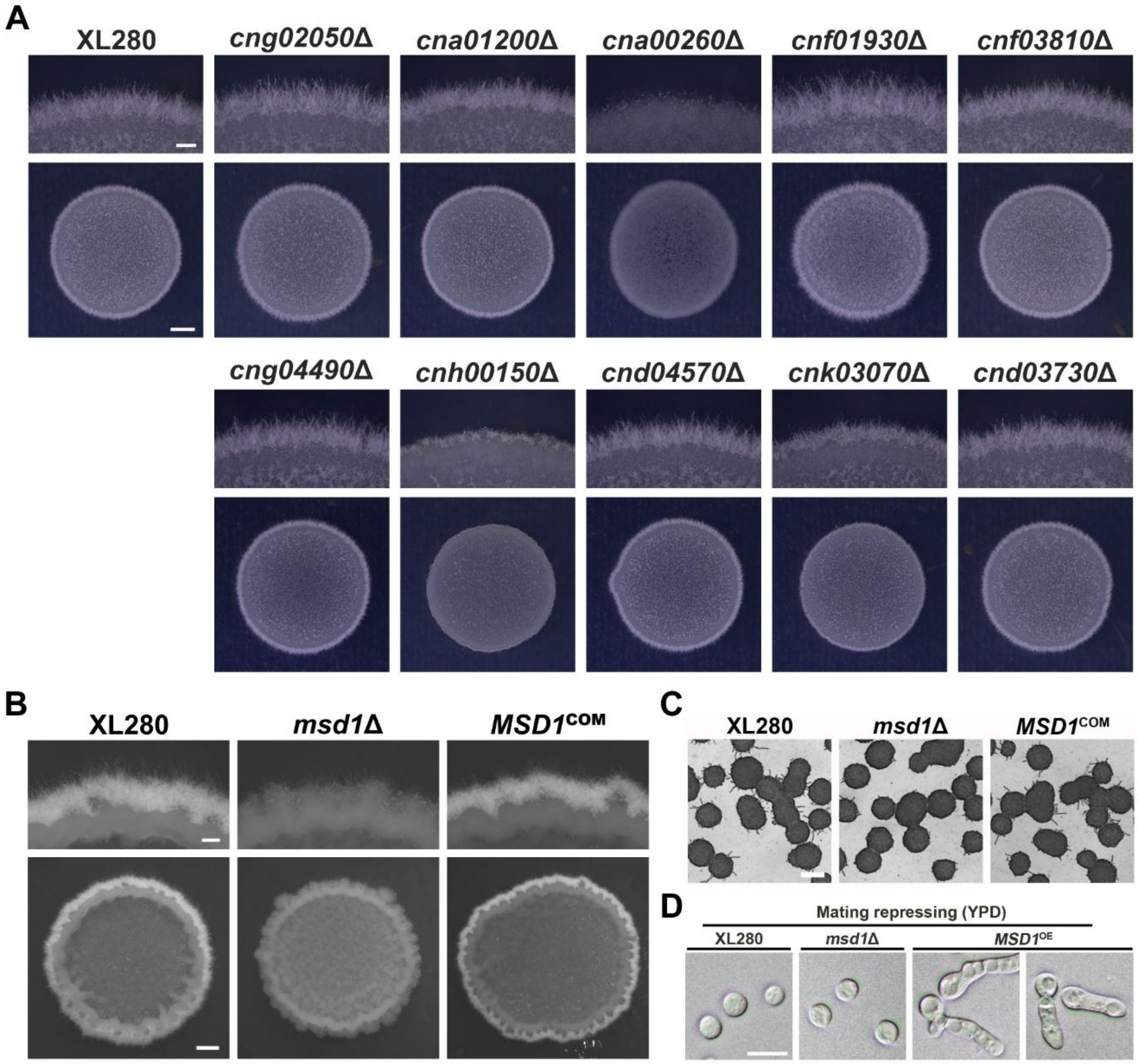

Fig. S3 Identification of Msd1 through Y1H screening and morphology-based phenotypic assay. (A) Unisexual filamentation assay of the TF knock-out mutants identified by the Y1H screen. The indicated strains were spotted onto V8 agar medium and incubated at 25℃ in the dark for three days. Scale bar, 0.2 mm for the upper panel, 1 mm for the bottom panel. **(B)** Complementation with a wild-type copy of *MSD1* (Gene ID: CNA00260) integrated into its native locus of the *msd1*Δ mutant background fully restored filamentation on V8 medium. The scale bars are consistent with those in panel A. (**C**) Disruption of *MSD1* impaired hyphal initiation during the early stage of unisexual development. The diluted (OD_{600}=0.02) cells of the indicated strains were spotted onto V8 medium and images were captured after incubation for 24 h. Scale bar, 100 μm. (**D**) Overexpression of *MSD1* under the control of the *GPD1* promoter drives yeast-to-hyphae transition even under mating-suppressing YPD medium. Scale bar, 10 μm.

Fig. S4

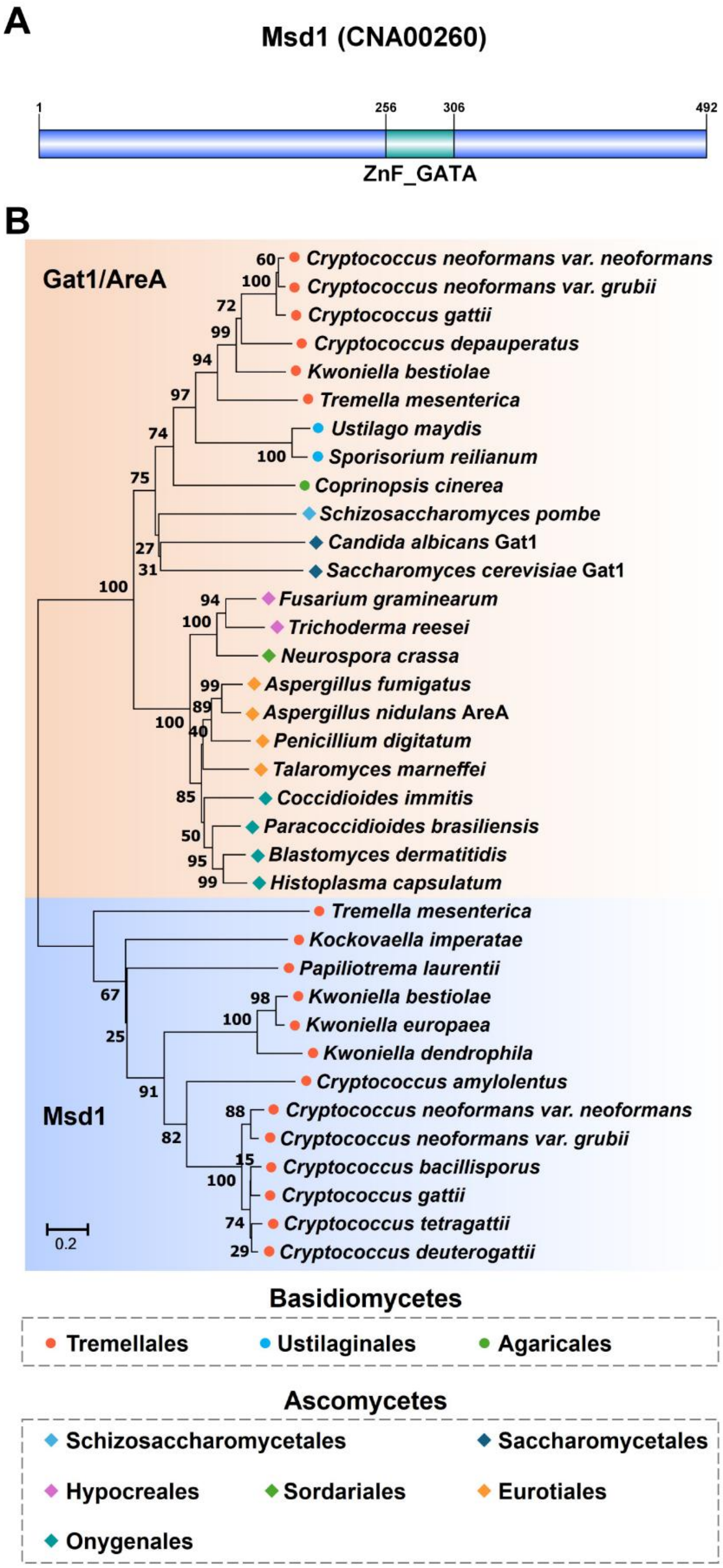

Fig. S4 *MSD1* encodes a GATA-type zinc finger protein, and its orthologs are exclusively distributed within the order *Tremellales* of the phylum Basidiomycota.

(**A**) Msd1 is predicted to carry a GATA-type zinc finger domain from amino acid 256 to 306. (**B**) Phylogenetic analysis of Msd1 and its closest paralog Gat1. Gat1 is an evolutionarily conserved transcription factor involved in nitrogen metabolism by controlling the expression of Nitrogen Catabolite Repression (NCR)-sensitive genes. Its orthologs are widely distributed in fungi, including the well-defined TF Gat1 in *Saccharomyces cerevisiae*/*Candida albicans* and AreA in *Aspergillus nidulans*. By contrast, the Msd1 orthologs are exclusively found in the order Tremellales within the Basidiomycetes. The Msd1 orthologs cluster together and are well separated from the branch of the Gat1/AreA orthologs. Sequence alignment was performed with ClustalW, and the neighbor-joining tree was generated with MEGA 7. Numbers on the tree branches represent the bootstrap support calculated per 1000 bootstrap replicates. The orders from basidiomycetes are marked with circle (●), while those from ascomycetes are marked with rhombus (♦).

Fig. S5

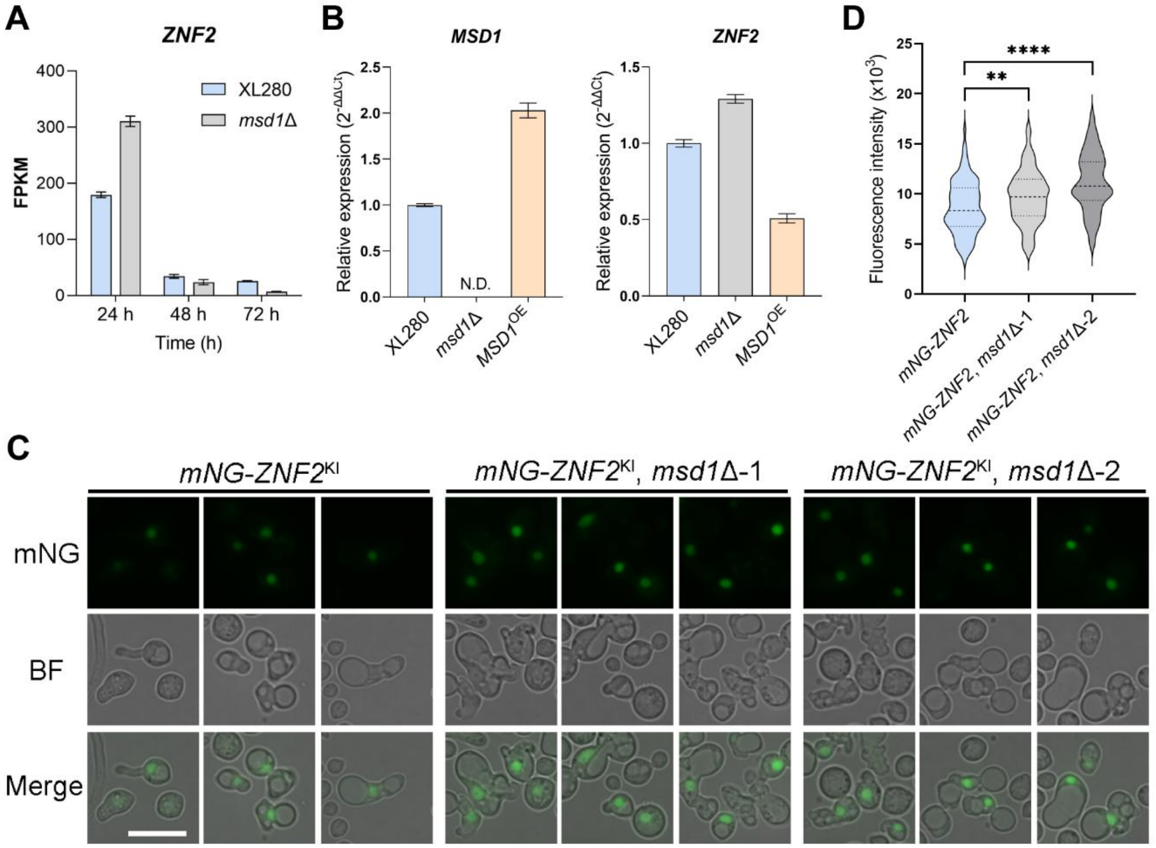

Fig. S5 Msd1 plays a repressive role in regulating *ZNF2* expression during the early stage of sexual development. (**A**) Transcriptional dynamics of *ZNF2* in XL280 and *msd1*Δ strains during different sexual development stages, as determined by RNA-Seq. FPKM of *ZNF2* from three biological replicates of each strain was shown. (**B**) RT qPCR-PCR was conducted to determine the *MSD1* and *ZNF2* transcript level in XL280, *msd1*Δ and *MSD1*^{OE} strains at the early stage of sexual development (V8, 24h). (**C**) Fluorescent signals of the natively tagged Znf2 in the *mNG-ZNF2*^{KI} and *mNG-ZNF2*^{KI}*msd1*Δ strains on V8 medium for 24h. mNG and BF indicate mNeonGreen and bright field respectively. Scale bar, 10 μm. (**D**) Quantification of the mNG-Znf2 fluorescence intensity of the indicated strains from panel B. One hundred cells with mNG-Znf2 expression signal of each strain were randomly selected for quantification. Statistical significance was determined by two-tailed Student's t-test (**, p<0.01; ****, p<0.0001).

Fig. S6

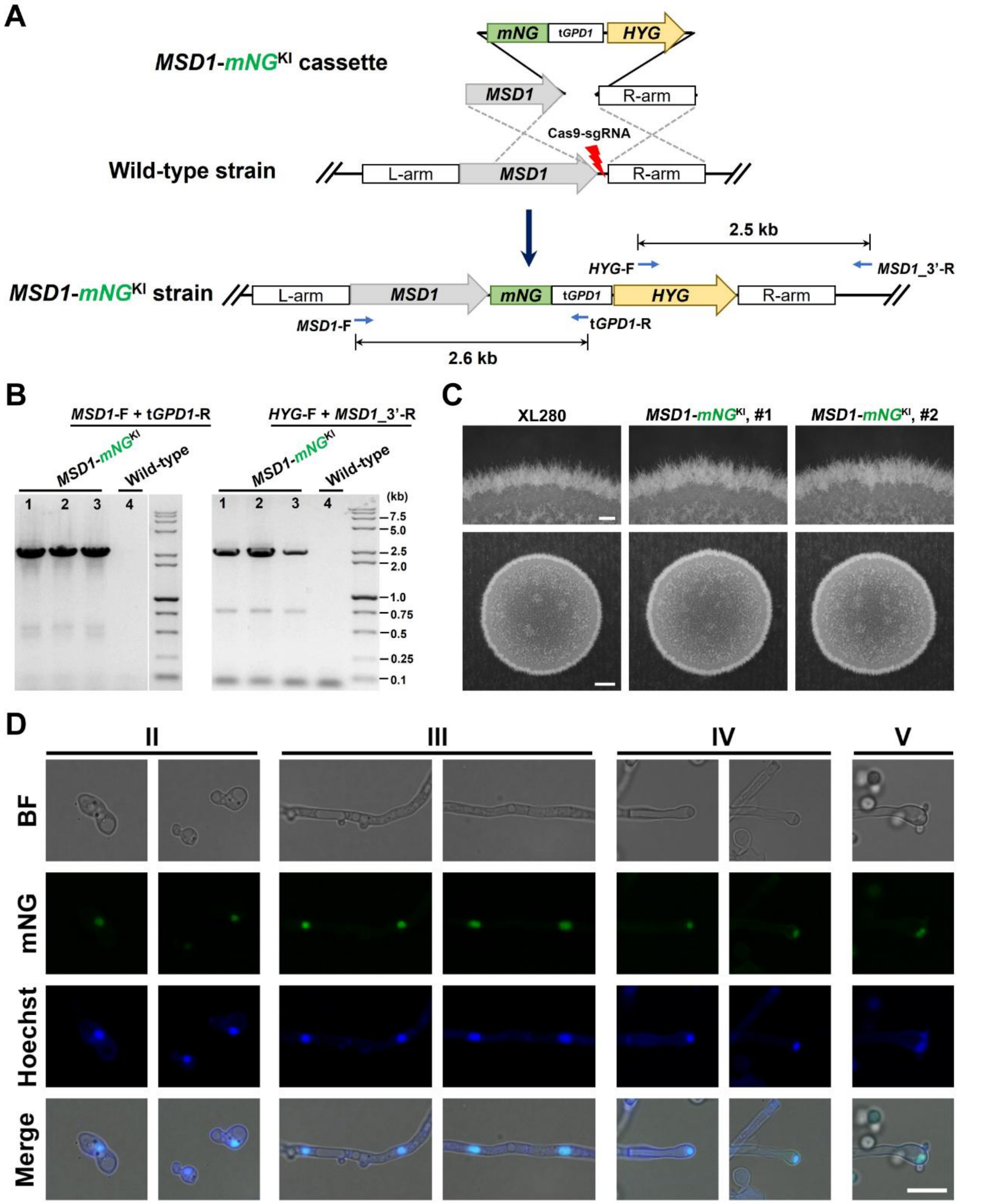

Fig. S6 Construction of the *MSD1-mNeonGreen* knock-in (*MSD1-mNG*^{KI}) strain. (**A**) Schematic diagram illustrating the construction process to generate the *MSD1-mNG*^{KI} strain. The knock-in cassette harbors a C-terminal mNeonGreen tagged *MSD1*, a HYG (hygromycin) selective marker and *MSD1* 3'-flanking sequence (R-arm). This DNA construct was transformed into the wild-type XL280 strain and integrated at the *MSD1* locus via the CRISPR-Cas9 system mediated double stranded break and homologous recombination-based repair. (**B**) Diagnostic PCR confirmation of the *MSD1-mNG*^{KI} strain. The primers used in PCR and the products size are indicated in panel A. Two independent

positive clones were shown. (**C**) Filamentation assay of the *MSD1-mNG*^{KI} strains during unisexual reproduction. The images were captured after three days of incubation on V8 medium at 25℃ in the dark. Scale bar, top panel 0.2 mm, bottom panel 1 mm. (**D**) Msd1-mNG localizes to the nucleus across the sexual development processes from stage II to stage V. The Msd1-mNG knock-in strain was cultured on V8 agar medium. Hoechst 33342 was used for nuclear DNA staining. Scale bar, 10 μm.

Fig. S7

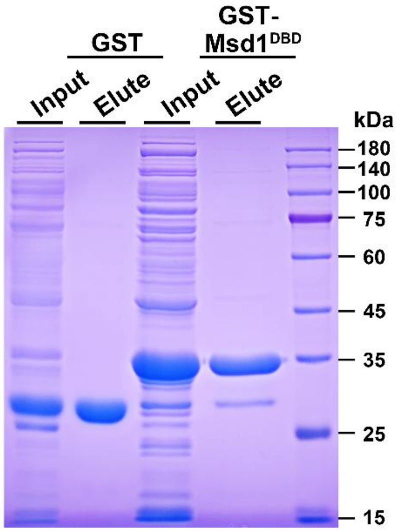

Fig. S7 Purification of GST and GST-Msd1^{DBD} from *E. coli*. Msd1 DNA-binding domain (DBD, aa 234~306) was fused to GST tag and expressed in *E. coli* Rosetta (DE3) strain. Input and elute of each sample were loaded for SDS-PAGE and Coomassie blue staining.

Fig. S8

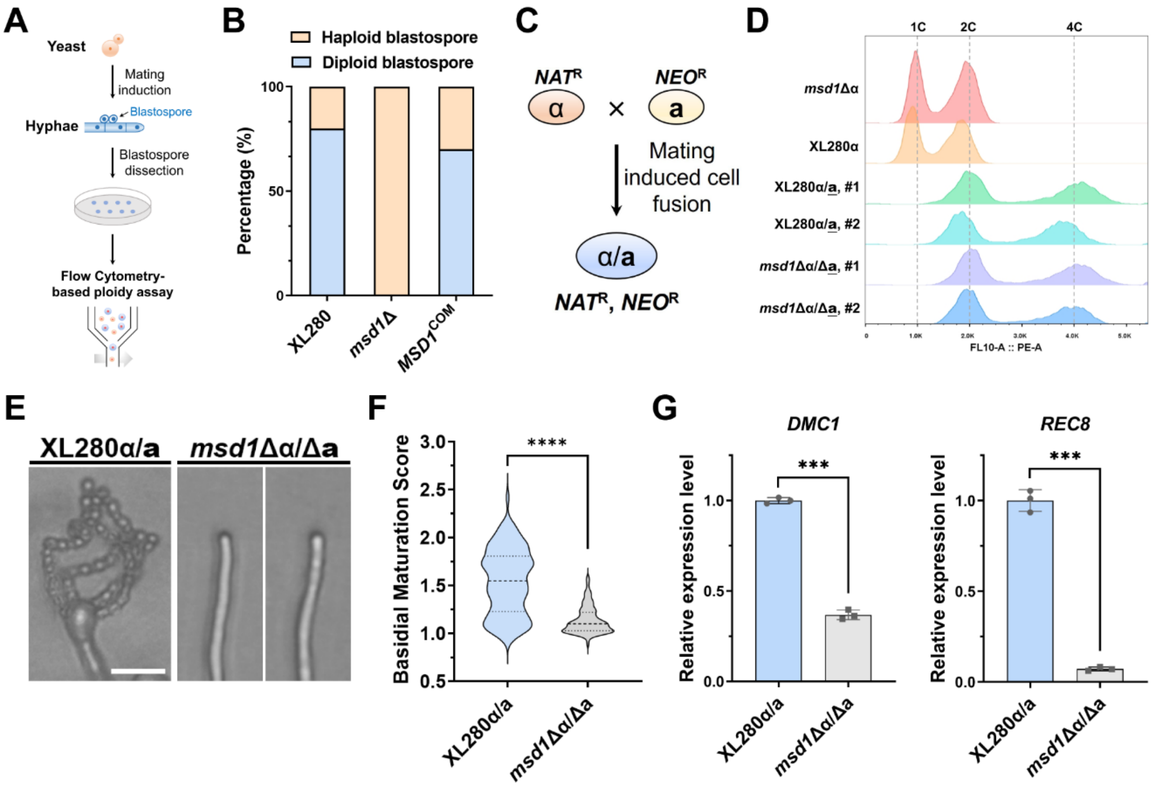

Fig. S8 Msd1 is required for the activation of meiosis and sporulation in the diploid background during sexual reproduction. (**A**) Schematic representation of blastospore dissection and subsequent ploidy analysis by Flow Cytometry. Blastospores dissected from each strain were stained with propidium iodide (PI) prior to ploidy analysis. (**B**) Ploidy assay of the blastospores dissected from WT, *msd1*Δ and *MSD1*^{com} strains via flow cytometry assay during unisexual reproduction. 50 blastospores of each strain were analyzed. **** p<0.0001, two-tailed Student's t-test. (**C**) Schematic diagram illustrating the generation of the diploid strains through cell-cell fusion under mating inducing condition. The resulting α/**a** diploid strains were isolated from the NAT+NEO double drug-resistant plate. (**D**) Ploidy validation of the XL280α/**a** and *msd1*Δα/*msd1*Δ**a** diploid strains via PI staining and flow cytometry assay. XL280α and *msd1*Δα strains were set up as haploid controls. 1C, 2C, and 4C represent the haploid, diploid, and tetraploid genomic states, respectively, as detected by flow cytometry. (**E**) Sporulation assay of the XL280α/**a** and *msd1*Δα/Δ**a** diploid strains on V8 medium for one month. Scale bar, 10 μm. (**F**) Basidial maturation assay of the hyphal population from the XL280α/**a** and *msd1*Δα/Δ**a** diploid strains during unisexual reproduction on V8 medium for two weeks. One-hundred hyphal tips of each strain were randomly selected for calculation. Statistical significance was determined by two-tailed Student's t-test (****, p<0.0001). (**G**) Relative transcript levels of *DMC1* and *REC8* in the XL280α/**a** and *msd1*Δα/Δ**a** diploid strains on V8 medium for 24 h. ***, p<0.001, two-tailed Student's t-test.

Fig. S9

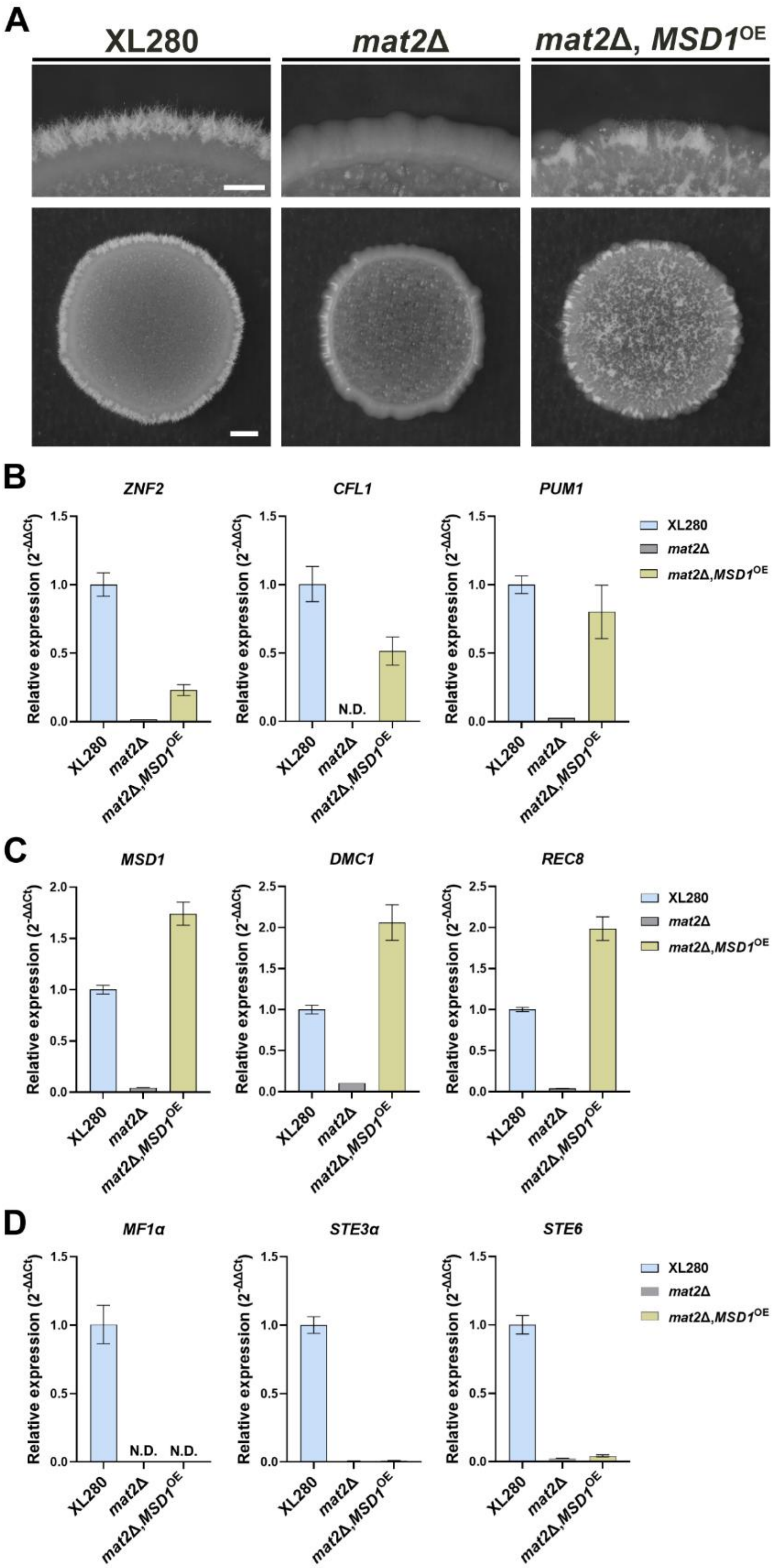

Fig. S9 Overexpression of *MSD1* in the *mat2*Δ mutant background restored filamentation and the expression of meiotic genes, but it did not restore the expression of the pheromone pathway genes. (**A**) Filamentation assay of the indicated

strains on V8 medium. The images were captured after three days of incubation on V8 medium at 25℃ in the dark. Scale bar, top panel 0.2 mm, bottom panel 1 mm. (**B~D**) RT-qPCR measuring the relative transcript levels of the filamentation genes (B), meiotic genes (C), or the pheromone pathway genes (D) in the indicated strains under mating-inducing condition (V8, 24h).

Fig. S10

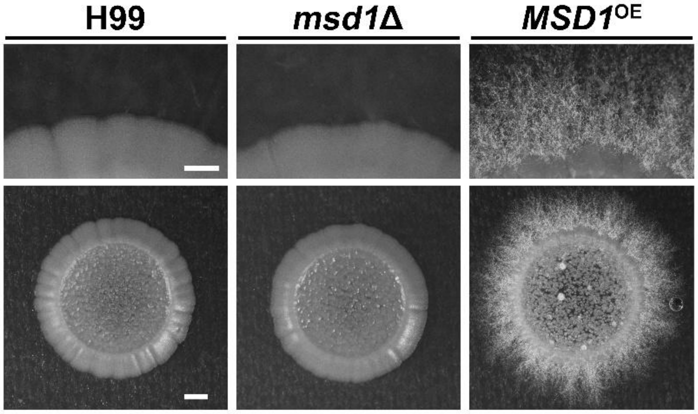

Fig. S10 Overexpressing *MSD1* in H99 background drives a robust self-filamentation under mating inducing condition. The indicated strains were cultured on V8 medium at 25℃ in the dark and the images were captured after five days of incubation. Scale bar, top panel 0.2 mm, bottom panel 1 mm.

Fig. S11

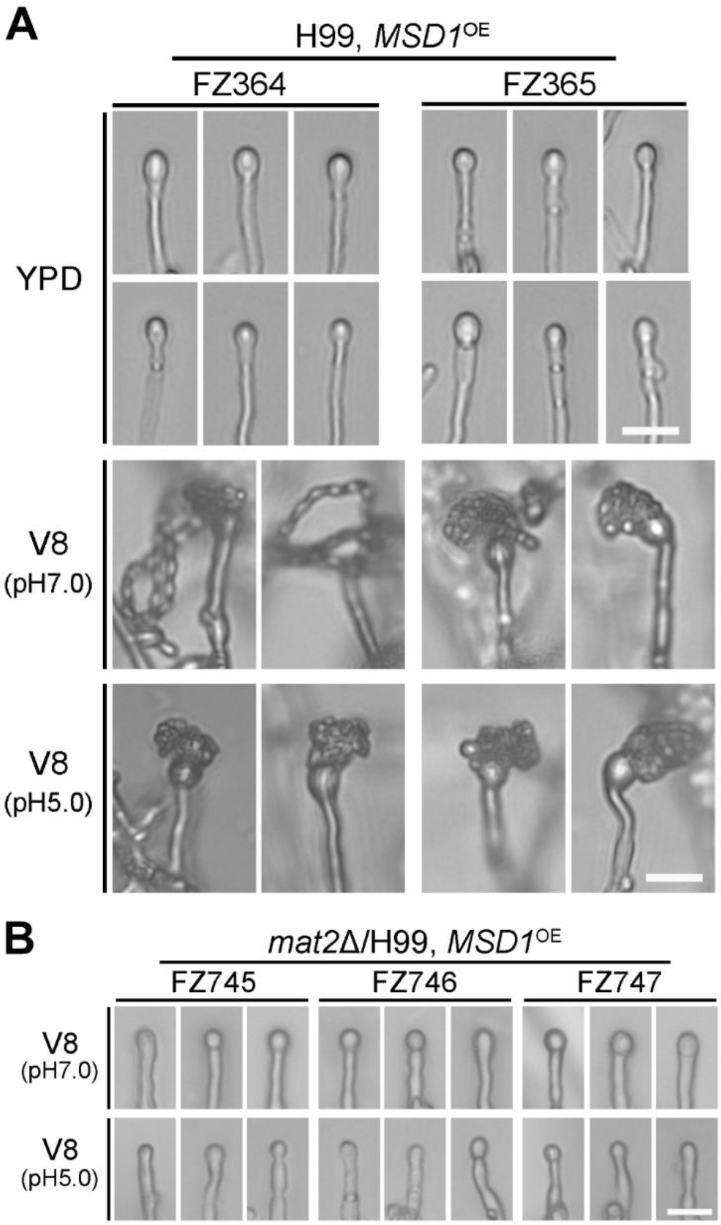

Fig. S11 *MSD1* overexpression drives sporogenesis in the serotype A reference strain H99 background. (**A**) Two representative *MSD1*^{OE} H99 strains were cultured on mating-repressing YPD and mating-inducing V8 (pH7.0 and pH5.0) media. Images were captured after two weeks of incubation. Scale bar, 10 μm. (**B**) Three representative *mat2*Δ/H99 *MSD1*^{OE} strains were cultured on V8 medium. Images were captured after four weeks of incubation. Scale bar, 10 μm.

Fig. S12

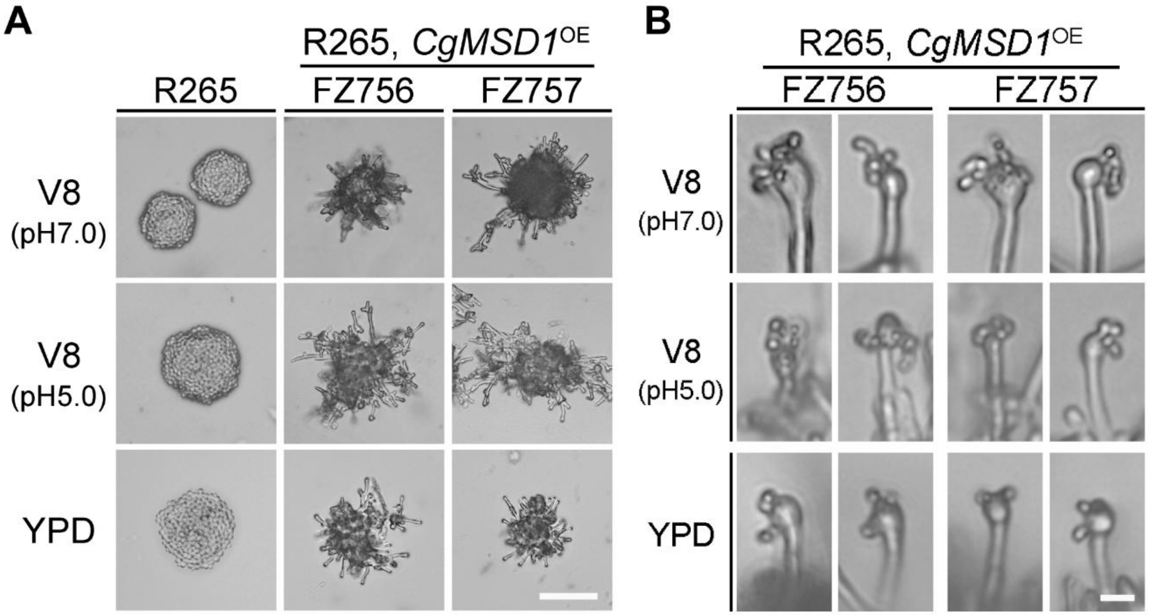

Fig. S12 Overexpression of *CgMSD1* in the *C. gattii* reference strain R265 is sufficient to drive sporogenesis under mating-inducing (V8) and mating-repressing (YPD) conditions. (**A**) Colony morphology of the wild-type R265 and two representative *CgMSD1*^{OE} strains cultured on YPD and V8 (pH7.0 and pH5.0) media for two days. Scale bar, 50 μm. (**B**) Spores were observed after one week incubating on V8 and YPD medium in the two representative *CgMSD1*^{OE} strains. Scale bar, 5 μm.

Fig. S13

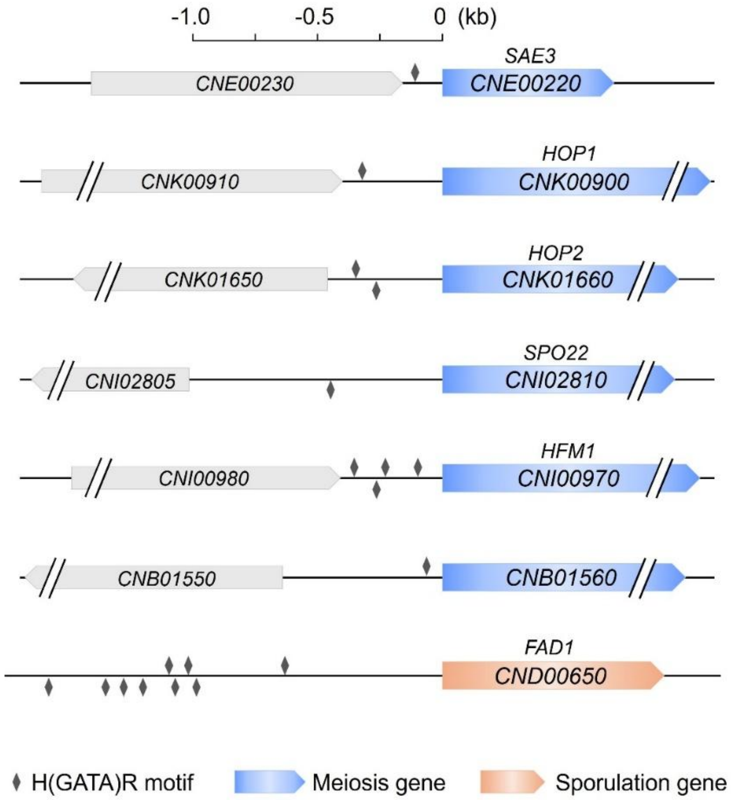

Fig. S13 Schematic diagram of the putative Msd1 binding motifs in the 1.5 kb upstream region of the meiosis and sporulation gene ORFs.

Fig. S14

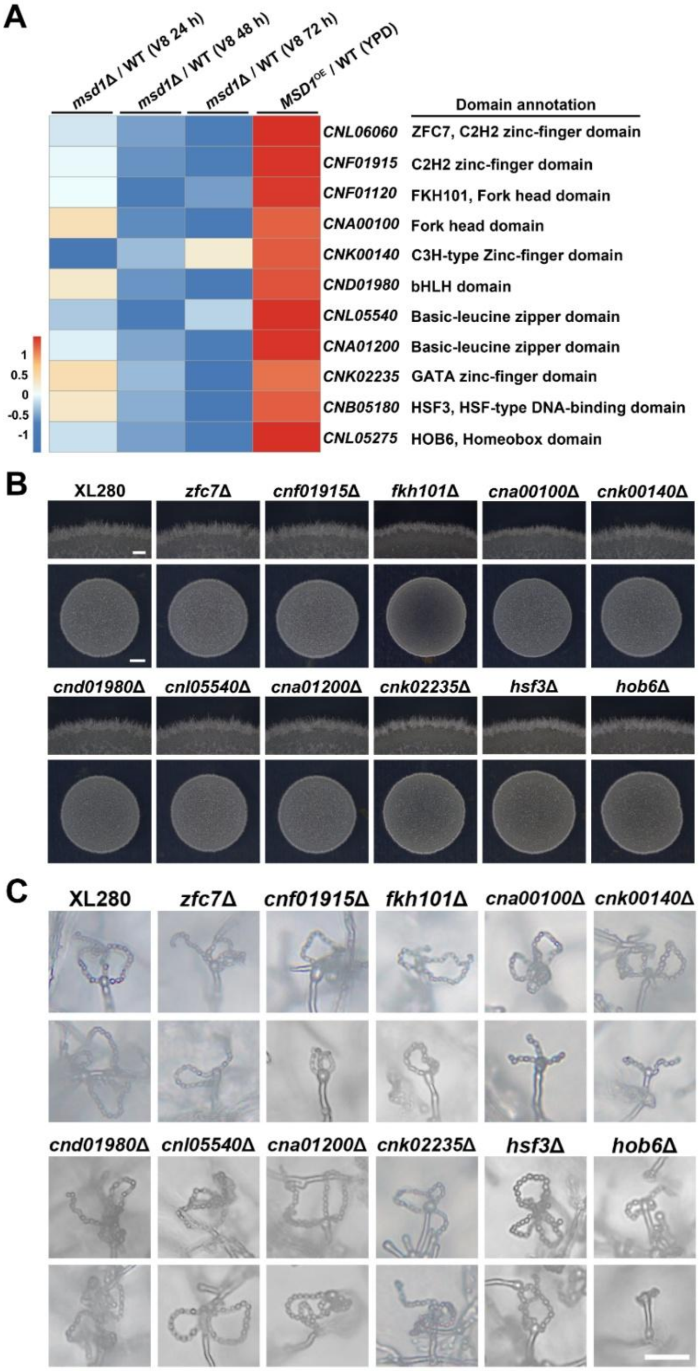

Fig. S14 Phenotypic analysis of the 11 putative Msd1 downstream transcription factor mutants. (**A**) Heatmap of the transcriptional changes of TF-encoding genes identified from Msd1 RNA-Seq data in the indicated groups. The DNA-binding domain of each gene was indicated at the right. Color bar indicated $\log_2$(fold change) value. (**B**) Filamentation assay of each TF gene deletion mutant under mating-inducing condition. The indicated strains were cultured on V8 medium and images were captured after three days of incubation at 25℃ in the dark. Scale bar, top panel 0.2 mm, bottom panel 1 mm. (**C**) Sporulation assay of each TF gene deletion mutant under mating inducing condition. Scale bar, 20 μm.

Fig. S15

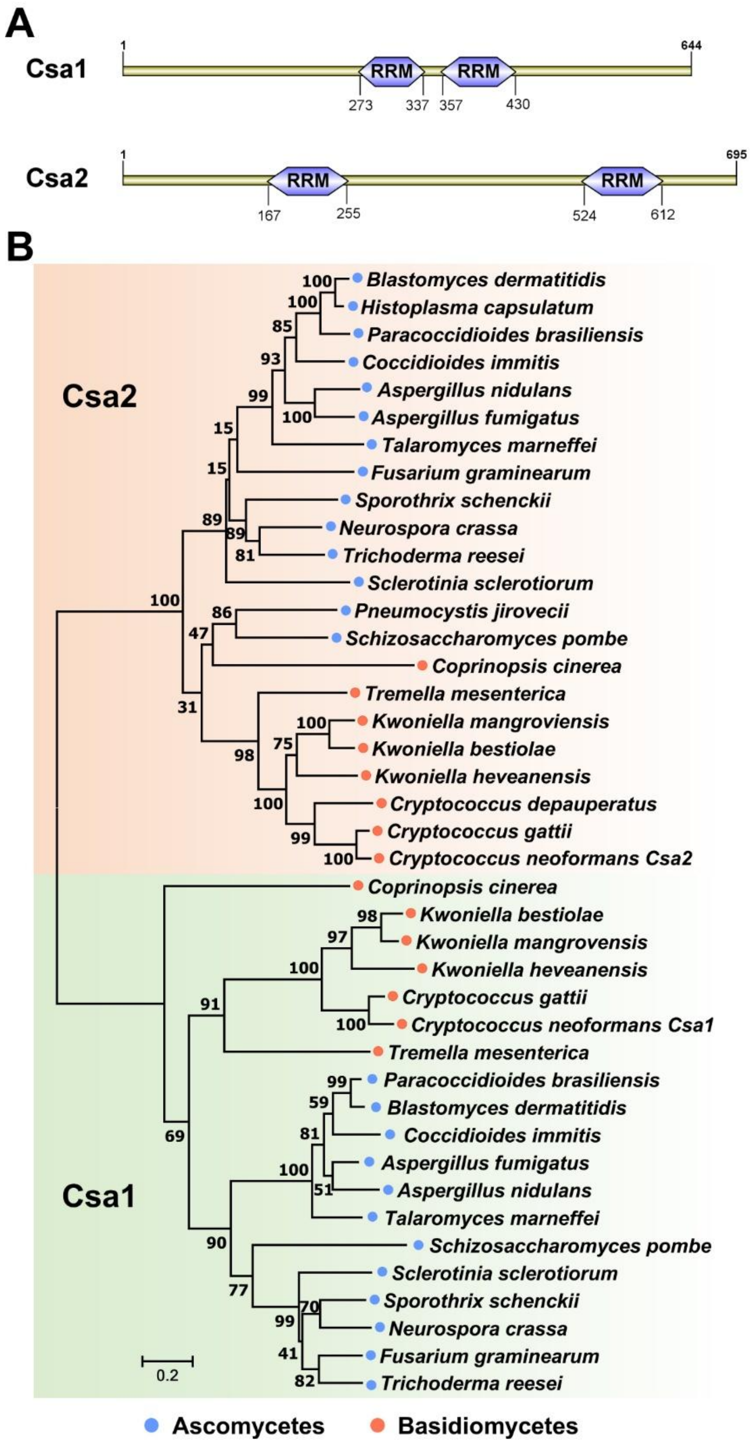

Fig. S15 Schematic illustration of the domain organization phylogenetic analysis of Csa1 and Csa2. (**A**) The RNA recognition motifs (RRMs) are indicated as bule hexagons. (**B**) Phylogenetic analysis of Csa1 and Csa2 and their orthologs from representative fungal species within ascomycetes and basidiomycetes. Sequence alignments were performed with ClustalW, and the neighbor-joining tree was generated with MEGA 7 software. Numbers on the tree branches represent the bootstrap support calculated per 1000 bootstrap replicates.

Fig. S16

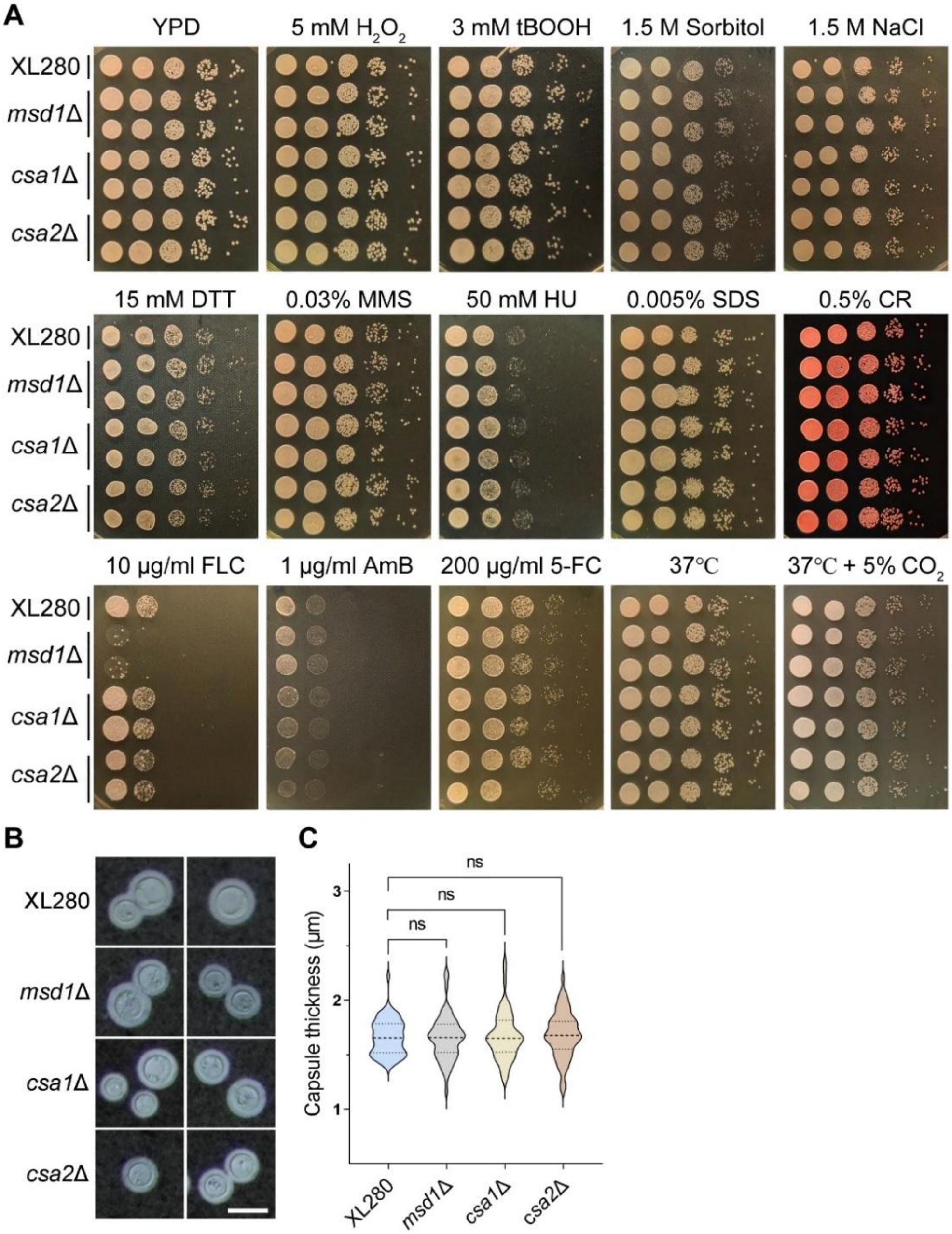

Fig. S16 Phenotypic assays of the *msd1*Δ, the *csa1*Δ and the *csa2*Δ mutants. (**A**) XL280, *msd1*Δ, *csa1*Δ and *csa2*Δ strains were ten times serially diluted and spotted onto agar media with different stressors. Two independent transformants of each mutant were used in the phenotypic assays. Images were captured after two days of incubation at 30℃ or 37℃. (**B**) Capsule formation of the wild-type XL280, the *msd1*Δ, the *csa1*Δ and the *csa2*Δ strains was examined after five days of incubation on RPMI + 5% CO_2 at 37℃. Scale bar, 10 µm. (**C**) Quantification of the capsule thickness of the indicated strains from panel B. Sixty cells from each strain were randomly selected for the measurement of capsule thickness. Statistical significance was determined by two-tailed Student's t-test (ns, p>0.05, not significant).

Fig. S17

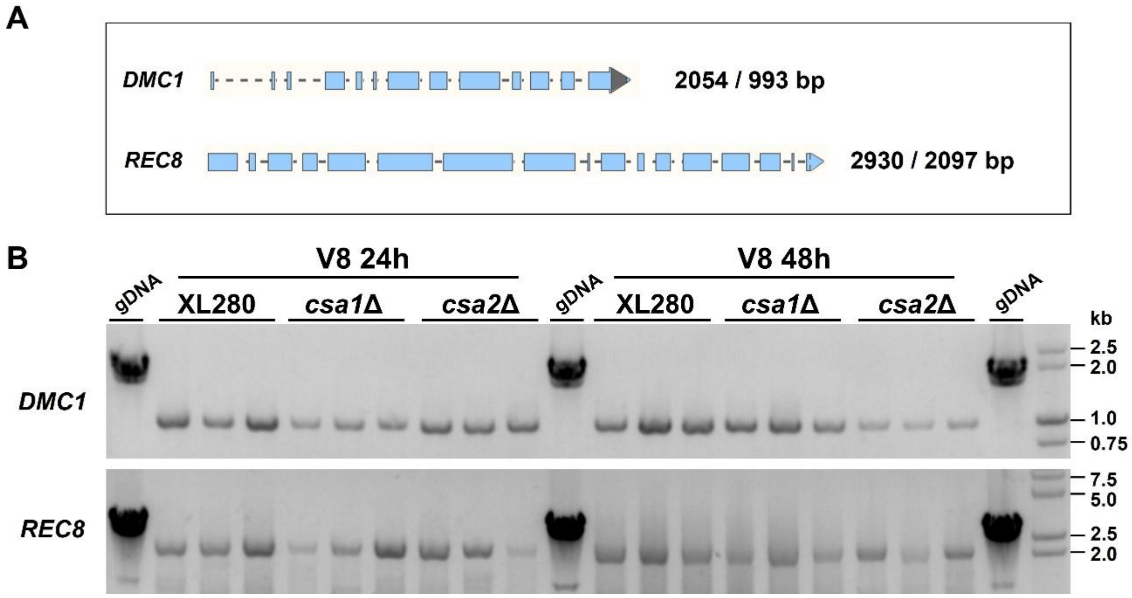

Fig. S17 Reverse transcription PCR (RT-PCR) assay confirmed that the splicing of *DMC1* and *REC8* occurs independently of Csa1 and Csa2. (**A**) Schematic representation of the gene structure of *DMC1* and *REC8*. The blue rectangles represent exons and the dashed lines indicate introns. The sizes of the open reading frame (ORF) and the spliced cDNA for each gene are indicated on the right side of the schematic diagram, respectively. (**B**) Reverse transcription PCR was performed using cDNA synthesized from RNA isolated from XL280, *csa1*Δ, and *csa2*Δ strains cultured on V8 medium at the indicated time points. Genomic DNA (gDNA) of the wild-type strain XL280 was used as the control. Three biological replicates of each strain were shown.

Tables

Table S1. List of the potential *ZNF2*-promoter binding proteins identified from the Y1H screen.

Bait	Gene ID	Annotation
P1, P3	CNG02050	CLR2, DNA binding protein
P1, P3	CNA01200	Basic-leucine zipper (bZIP) domain transcription factor
P1, P3	**CNA00260**	**GATA-type zinc finger domain protein**
P1	CNF01930	STB4, putative transcription factor
P1	CNF03810	C3H1-type Zinc finger protein
P1	CNG04490	SET and MYND domain protein
P3	CNH00150	APSES-type HTH DNA-binding domain protein
P3	CND04570	C3H1-type Zinc finger protein, Nucleoporin NUP42
P3	CNK03070	C2H2 zinc finger protein Zas1A
P3	CND03730	Fungal specific transcription factor

Table S2. Strains and plasmids used in this study.

Strains

Strain Name	Genotype	Annotation	Reference or source
XL280α	MATα, wild type *C. neoformans* serotype D strain	Wild type	PMID: 17112316(1)
XL280a	MATa, wild type *C. neoformans* serotype D strain	Wild type	PMID: 23670559(2)
H99	MATα, wild type serotype A strain	Wild type	PMID: 12933823(3)
R265	MATα, wild type *C. gattii* strain	Wild type	PMID: 10512666(4)
LW781	MATα, P_{DMC1}-*DMC1*-*mCherry*-HYG	*DMC1-mCherry* reporter strain	PMID: 24901238(5)
FZ61	MATα, *msd1*::NAT	*MSD1* deletion strain in XL280α background	This study
FZ158	MATα, *msd1*::NEO	*MSD1* deletion strain in H99α background	This study
FZ75	MATα, *msd1*::NAT, P_{GPD1}-*MSD1*-HYG	*MSD1* overexpression strain in XL280 background	This study
FZ214	MATα, P_{ZNF2}-*mNeonGreen*-*ZNF2*-*NEO*^{KI}	*mNeonGreen-ZNF2* knock-in strain	This study
FZ130	MATα, *msd1*::NAT, P_{MSD1}-mNeonGreen-*MSD1*-NEO	*MSD1* complemental strain	This study
FZ248	MATα, P_{MSD1}-*MSD1*-mNeonGreen-HYG^{KI}	*MSD1*-mNeonGreen knock-in strain	This study

FZ189	MATα, P_{DMC1}-*DMC1-mCherry*-HYG, *msd1*::NAT	*MSD1* deletion strain in the *DMC1-mCherry* reporter strain	This study
FZ289	MATα, P_{DMC1}-*DMC1*-mCherry-HYG, P_{GPD1}-*MSD1-mNeonGreen-NEO*	*MSD1-mNG* overexpression strain in the *DMC1-mCherry* reporter strain background	This study
FZ364/FZ365	MATα, P_{GPD1}-*MSD1(A)-HYG*	*MSD1* overexpression strain in H99α	This study
FZ407	MATα/a, *msd1*::NATα, *msd1*::NEO**a**	*msd1*Δα/*msd1*Δ**a** diploid strain	This study
FZ401	MATα/**a**, XL280::NEOα, XL280::*NAT***a**	XL280α/**a** diploid strain	This study
FZ51	MATα, P_{CTR4}-*3FLAG-ZNF2-NEO*	*ZNF2* overexpression strain in XL280α	This study
FZ278	MATα, *msd1*::*NAT*, P_{GPD1}-*MSD1-mNeonGreen-HYG*	Msd1-mNeonGreen overexpression strain in XL280α	This study
FZ152	MATα, *mat2*::NAT	*MAT2* deletion strain in XL280α	This study
FZ169	MATα, *mat2*::*NAT*, P_{GPD1}-*MSD1-HYG*	Overexpressing *MSD1* in *mat2*Δ/XL280 strain background	This study
XP1397	MATα, *csa1::NAT*	*CSA1* deletion strain in XL280 background	This study
XP1399	MATα, *csa2::NEO*	*CSA2* deletion strain in XL280 background	This study
FZ699	MATα, *csa1::NAT*, P_{GPD1}-*MSD1-mNeonGreen-HYG*	*MSD1-mNG* overexpression strain in *csa1*Δ background	This study
FZ703	MATα, *csa2::NEO*, P_{GPD1}-*MSD1-mNeonGreen-HYG*	*MSD1-mNG* overexpression strain in *csa2*Δ background	This study
FZ745/FZ746/ FZ747	MATα, *mat2*::*NEO*, P_{GPD1}-*MSD1-HYG*	Overexpressing *MSD1* in *mat2*Δ/H99 strain background	This study
FZ756/FZ757	MATα, P_{GPD1}-*CgMSD1-HYG*	Overexpressing *CgMDS1* in R265	This study
Y187	*MATα, ura3-52, his3-200, ade2-101, trp1-901, leu2-3, 112, gal4Δ, gal80Δ, met–, URA3 : : GAL1*$_{UAS}$*–Gal1*$_{TATA}$*–LacZ, MEL1*	*S. cerevisiae* strain used for yeast one-hybrid assay	Clontech
Y2H Gold	*MATa, trp1-901, leu2-3, 112, ura3-52, his3-200, gal4Δ, gal80Δ, LYS2::GAL1*$_{UAS}$*–Gal1*$_{TATA}$*–His3, GAL2*$_{UAS}$*–Gal2*$_{TATA}$*–Ade2, URA3::MEL1*$_{UAS}$*–Mel1*$_{TATA}$ *AUR1-C MEL1*	*S. cerevisiae* strain used for yeast two-hybrid assay	Clontech

Plasmids

Plasmid name	Annotation	Reference or source

p*RS1*	Y1H bait plamid	PMID: 28378498(6)
pRS1-ZNF2pr-1	Y1H bait plamid	This study
pRS1-ZNF2pr-3	Y1H bait plamid	This study
pGADT7-MSD1	Y1H prey plasmid	This study
pZNF2-mNeonGreen-ZNF2-KI-NEO	mNG-ZNF2 knock-in plasmid	This study
pCFL1-CFL1-mCherry-NEO	Cfl1-mCherry reporter plasmid	PMID: 22737071(7)
pDMC1-DMC1-mCherry-HYG	Dmc1-mCherry reporter plasmid	PMID: 24901238(5)
pXL1	Overexpression plasmid with GPD1 promoter	PMID: 22737071(7)
pXL1-MSD1-HYG	*MSD1* overexpression plasmid under control of *GPD1* promoter	This study
pFZ10	mNeonGreen tag plasmid for gene overexpression	This study
pFZ10-MSD1-HYG (pGPD1-MSD1-mNeonGreen-HYG)	Msd1-mNG overexpression plasmid under control of *GPD1* promoter	This study
pET-GST-6His	Recombinant protein expression plasmid	This study
pET-GST-Msd1DBD-6His	GST-Msd1DBD Recombinant protein expression plasmid	This study
pGBKT7	Y2H plasmid	Clontech
pGADT7	Y2H plasmid	Clontech
pGBKT7-CSA1	Y2H plasmid	This study
pGADT7-CSA2	Y2H plasmid	This study
pGBKT7-Lam	Y2H plasmid	Clontech
pGBKT7-p53	Y2H plasmid	Clontech
pGADT7-T	Y2H plasmid	Clontech

Table S3. Primer used in this study.

Primer code	Primer sequence	Annotation
ZFL-1	GTAAAACGACGGCCAGT	M13F
ZFL-2	ACAGGAAACAGCTATGAC	M13R
ZFL-3	TAATACGACTCACTATAGGG	T7
ZFL-4	AGATGGTGCACGATGCACAG	3’AD
ZFL-5	CATCCATCTCGCCTTGTCC	NAT-in F
ZFL-6	AAATTCTGTAGCAAGACCCATC	NAT-in R
ZFL-9	ACTCCCTGGTCCCATCCCT	CnU6 promoter Far-F
ZFL-10	AACTGAGATACCTACAGCGTGAG	sgRNA scaffold Far-R

ZFL-11	CCATCGATTTGCATTAGAACTAAAAACAAAGCA	U6 promoter F
ZFL-12	CCGCTCGAGTAAAACAAAAAAGCACCGAC	gRNA-R
ZFL-13	GATGTTTTGGGTCAGAGAACAACAGTATACCCTGCC GGTG	gRNA(XL280_SH3)-U6 R
ZFL-14	GTTCTCTGACCCAAAACATCGTTTTAGAGCTAGAAA TAGCAAGTT	gRNA(XL280_SH3)-Scaffold F
ZFL-17	CGAAGGATGGTTGTCGCTC	SH3 (D) screening F
ZFL-18	GTATCGTCTTGCTCTTCATTCC	SH3 (D) screening R
ZFL-19	GGAGACCTCAACCCAGACAACT	Cas9 screening F
ZFL-20	CCCTCCTCGATACGCTTCATAC	Cas9 screening R
ZFL-21	ACCAGATTACGCTCATATGAGCAACCAAAAAGAACA TCAG	MSD1-NdeI F
ZFL-22	GCTCGAGCTCGATGGATCCTTATGCAGAGTCATTAT CCTCCTGT	MSD1-BamHI R
ZFL-23	GAACAAAAGCTGGGTACCTTACGCAGGGCGAAACT TTGTG	Pznf2-944 F (P1)
ZFL-24	ATCCCTCGAGGGCCCGTGTTGGAGACGCTCGGAGA TG	Pznf2-222 R (P1)
ZFL-25	GAACAAAAGCTGGGTACCCCTTCTACTCAAGTGCAG CCTTTC	Pznf2-2177 F (P3)
ZFL-26	ATCCCTCGAGGGCCCGGAGACTTGATACAGCCCTC GTT	Pznf2-811R (P2)
ZFL-27	GAACAAAAGCTGGGTACCGTTGCGAACAGGGTGGG AG	Pznf2-4331 F (P4)
ZFL-28	ATCCCTCGAGGGCCCGCTCAAATGCCTCTATGTTCA GC	Pznf2-2963R (P4)
ZFL-29	AGTGTCCGATGATGGCAAATGA	MSD1-Far-L F
ZFL-30	CGTGATAAACTCATGCCTACCC	MSD1-nest F
ZFL-31	TTGGCACTGGCCGTCGTTTTACGCTCACCCTTGTGA TACTCCTG	MSD1-LR with M13F
ZFL-32	TCATGTCATAGCTGTTTCCTGTTCTGCATAAGGTCC CACAGTAC	MSD1-RF with M13R
ZFL-33	GCTCAATAGTCTCGTGCTGATA	MSD1-nest R
ZFL-34	AATAGCGACTCACTGAGCTTGA	MSD1-R-outside R
ZFL-35	CCAGAAATCTTCACCCCTGTAACAGTATACCCTGCC GGTG	gRNA(MSD1)-U6R
ZFL-36	ACAGGGGTGAAGATTTCTGGGTTTTAGAGCTAGAAA TAGCAAGTT	gRNA(MSD1)-ScaffoldF
ZFL-48	ATCCCTCGAGGGCCCGACGCATTTCGCAGGGTGAT TGG	Pznf2-1119R (P3)
ZFL-49	GAACAAAAGCTGGGTACCCCGGCACCCTTCCTTTGT TCCACA	Pznf2-1539 F (P2)
ZFL-43	GGCGGTGGCTCTGGGCCGGCCAGCAACCAAAAAG AACATCAGTAT	MSD1-FseI F
ZFL-44	TACTGTAACCCTTAATTAATTATGCAGAGTCATTATC CTCCTG	MSD1-PacI R
ZFL-63	CTCATGCCCACCCACTGCCTCT	MSD1_H99 Far-L F
ZFL-64	TGCCACGAGGCAATCTACAGC	MSD1_H99 nest F

ZFL-65	TTGGCACTGGCCGTCGTTTTAGGTGACCTCCCAATA CTGATG	MSD1_H99 L-R with M13F
ZFL-66	TCATGTCATAGCTGTTTCCTGTTGACTCCTACTACC GGACAA	MSD1_H99 RF with M13R
ZFL-67	AAGATTCACATCCTGGGC	MSD1_H99 nest R
ZFL-68	CGCAAACCTCAGACGTAAGC	MSD1_H99 outside R
ZFL-69	TCTTTCTCCCGTAGCCGTACAACAGTATACCCTGCC GGTG	gRNA(MSD1)-U6R
ZFL-70	GTACGGCTACGGGAGAAAGAGTTTTAGAGCTAGAA ATAGCAAGTT	gRNA(MSD1)-ScaffoldF
ZFL-128	AATTCGAGCTCGGTACCCGGGCGTGATAAACTCAT GCCTACCC	mNG-MSD1-com L-F
ZFL-129	CTCGCCCTTGGACACCATTGGAAAAGCTCCTACTAC GTCTGC	mNG-MSD1-com L-R
ZFL-130	TGCATGCCTGCAGGTCGACTCTAGAGCTCAATAGTC TCGTGCTGATA	mNG-MSD1-com R-arm R
ZFL-131	ACTGGCGGCCGTTACTAGTGGATCCCGCCATGTTT CCACCAGTTT	mNG-MSD1-com R_arm F
ZFL-132	ATCAAAGCTCTACCGACACCAACAGTATACCCTGCC GGTG	gRNA(NAT)-U6R
ZFL-133	GGTGTCGGTAGAGCTTTGATGTTTTAGAGCTAGAAA TAGCAAGTT	gRNA(NAT)-ScaffoldF
ZFL-685	ATTATCACAATGTCTGATAACGAAGA	DMC1-ATG F
ZFL-686	TTAGCTTGGGTCTTCCCAGC	DMC1-TAA R
ZFL-687	ATGGCCACGCTGGGACCTC	REC8-ATG F
ZFL-688	TTACGGATTTTTGACAGCAAAATG	REC8-TAA R
ZFL-71	TCCCAACCTATCTTCTTCTGCT	MSD1 qPCR-F
ZFL-72	GCGTCATACTCGGACTGCTC	MSD1 qPCR-R
ZFL-73	CCGTGACTGCCGTTGGCTTA	ZNF2 qPCR-F
ZFL-74	CATCGTCGCTATCGTCCTCTG	ZNF2 qPCR-R
ZFL-75	CTCAAACCTCATCTCCGTCAC	MAT2 qPCR-F
ZFL-76	CGGGTTGCTGGTATGTTGC	MAT2 qPCR-R
ZFL-77	AGGCAAGCGAACTATTCAGGC	CFL1 qPCR-F
ZFL-78	TGACCCTCGCCAGTGGAAG	CFL1 qPCR-R
ZFL-183	TTCCGTCAAGACTTCAATCCTAC	PUM1 qPCR-F
ZFL-184	CAACAACGCACTTCTAACTACTCC	PUM1 qPCR-R
ZFL-185	AACGTCGTCTACATAACAACCG	DMC1 qPCR-F
ZFL-186	CAACTTTACCGCTACCACCA	DMC1 qPCR-R
ZFL-284	TCCCATCTCACTCATGTACCCA	REC8 qPCR-F
ZFL-285	GCTCTTGAACGTCAATATCCTTT	REC8 qPCR-R
ZFL-286	ATCTTCACCACCTTCACTTCT	MF1α qPCR-F
ZFL-287	CTAGGCGATGACACAAAGG	MF1α qPCR-R
ZFL-288	TAGCGGAGCGGACTGGAAAGA	STE3α qPCR-F

ZFL-289	CTCGACCGAGACGGCAATCATTA	STE3α qPCR-R
ZFL-290	GCGAATCCACCACCGAATCAATC	STE6α qPCR-F
ZFL-291	CGACGACTGCAACGCACTCT	STE6α qPCR-R
ZFL-239	GGGCTGTGATAGGGCTGAGA	DMC1 ChIP qPCR-F
ZFL-240	CGCTCGTCGTAATCAAAGTG	DMC1 ChIP qPCR-R
ZFL-479	TCCTTTCCCGTCGCATTAGTTT	REC8 ChIP qPCR-F
ZFL-480	GACTGACGATCCCTTCTTGCTC	REC8 ChIP qPCR-R
ZFL-481	ACGAACCAGTCATCTGCGTCA	CSA1 ChIP qPCR-F
ZFL-482	AATCATCTAAATGTGGGAAACG	CSA1 ChIP qPCR-R
ZFL-483	GGTGGAATCAGCACGGATACAG	CSA2 ChIP qPCR-F
ZFL-484	AAGAATCGCGTCTTTAATTTCCAG	CSA2 ChIP qPCR-R
ZFL-239	GGGCTGTGATAGGGCTGAGA	DMC1 ChIP qPCR-F
ZFL-240	CGCTCGTCGTAATCAAAGTG	DMC1 ChIP qPCR-R
ZFL-479	TCCTTTCCCGTCGCATTAGTTT	REC8 ChIP qPCR-F
ZFL-480	GACTGACGATCCCTTCTTGCTC	REC8 ChIP qPCR-R
ZFL-481	ACGAACCAGTCATCTGCGTCA	CSA1 ChIP qPCR-F
ZFL-482	AATCATCTAAATGTGGGAAACG	CSA1 ChIP qPCR-R
ZFL-483	GGTGGAATCAGCACGGATACAG	CSA2 ChIP qPCR-F
ZFL-484	AAGAATCGCGTCTTTAATTTCCAG	CSA2 ChIP qPCR-R

Methods

Strains and plasmids construction

To construct the yeast bait strains for Y1H screening, the designed *ZNF2* promoter regions (Pr_{ZNF2}-P1, Pr_{ZNF2}-P2 and Pr_{ZNF2}-P3) were amplified from XL280 genomic DNA, and then cloned into the *Kpn*I/*Apa*I site of the plasmid pRS1, resulting in the creation of bait plasmids pRS1-*ZNF2*-P1, pRS1-*ZNF2*-P2 and pRS1-*ZNF2*-P3 containing the bait fragment upstream of the *GAL1* core promoter and the AbA resistant gene *AUR1-C* as previous described (6). After confirmation of the bait plasmids by restriction enzyme digestion and Sanger sequencing, these bait plasmids were linearized with restriction endonuclease *Bsu36*I and then transformed into the yeast Y187 strain to generate the bait yeast strains. The genotype of the bait strains was confirmed by diagnostic PCR. Potential auto-activation of these three baits in yeast were tested on SD/-Trp/AbA selective plate prior to their transformation with the cryptococcal cDNA library.

The *Cryptococcus* knock-out strains were constructed via TRACE (Transient CRISPR-Cas9 coupled with Electroporation) method as described in our previous publications (8). Briefly, approximately 1.0 kb of 5' and 3' flanking sequences of the target gene ORF were amplified from the parental strain genomic DNA and fused with two split parts of the NAT dominant drug marker by overlapping PCR respectively. Meanwhile, the DNA construct encoding the guide RNA (sgRNA) was generated via fusing the U6 promoter, 20-bp target sequence, and the sgRNA scaffold together as we described previously (8). The Cas9 expression cassette was amplified from the plasmid pXL1-CAS9-HYG with the universal primer pair M13F/M13R. All of these DNA fragments were purified by using a commercial DNA purification kit (D6492, OMEGA Biotek). Subsequently, the knock-out construct, the corresponding sgRNA encoding fragment, and the *CAS9* cassette were mixed together and transformed into the recipient strain via electroporation as we described previously (8). Transformants were screened on YPD plate supplemented with corresponding selective drug, and the genotype of randomly picked transformants was confirmed by diagnostic PCR.

To complement the knockout mutants, the ORF plus approximately 1.5 kb 5'-flanking sequence were amplified from the wild-type strain and cloned into *Xba*I/*Pac*I sites of the pXL1 plasmid. For gene overexpression, the target gene ORF was amplified from wild-type genomic DNA and cloned into the *Fse*I/*Pac*I sites of the pXL1 plasmid. To construct the *MSD1-mNG*^{OE} strain where overexpressed *MSD1* was labeled with C-terminal mNeonGreen tag, the *MSD1* ORF amplified from wild-type genomic DNA was cloned into the *Apa*I/*Asc*I sites of the pFZ10 (pGPD1-ApaI-AscI-mNeonGreen-HYG) plasmid to obtain the pFZ10-*MSD1*-HYG plasmid. All of these plasmids were constructed by using a one-step cloning kit (C112, Vazyme) and confirmed by restriction enzyme digestion and Sanger sequencing. The complementation and the overexpression constructs were then amplified from these plasmids using universal primer pair M13F/M13R, combined with *CAS9* and *SH3* sgRNA fragments targeting the safe haven 3 locus (9), and transformed into the knock-out strains via electroporation.

To construct the mNeonGreen-*ZNF2* knock-in strain, the *ZNF2* ORF was initially cloned into *Fse*I/*Pac*I site of the plasmid pFZ1 (p*CTR4*-mNG-NEO) to generate p*CTR4*-mNG-*ZNF2*-NEO plasmid. The *CTR4* promoter was then removed via *Apa*I/*Sma*I digestion and replaced with the 1.5 kb of *ZNF2* 5'-flanking sequence. Subsequently, a 1.2 kb of *ZNF2* 3'-flanking sequence was cloned into the *Spe*I/*Sal*I sites to generate the final

knock-in plasmid. The knock-in construct was amplified from these two plasmids with the universal primer pair M13F/M13R, combined with *CAS9* and sgRNA fragment targeting selective marker gene *NAT*, and then transformed into the *znf*2Δ mutant via electroporation. To construct the *MSD1-mNG* knock-in strain, a 1.2 kb *MSD1* 3'-flanking sequence was cloned into *BamH*I/*Xba*I sites of the aforementioned plasmid p*FZ10-MSD1-HYG*, resulting in the final *MSD1-mNG*^{KI} plasmid. The *MSD1-mNG* knock-in construct was amplified with primer pair MSD1-inF/M13R from this plasmid and introduced into the wild-type XL280 strain to generate the *MSD1-mNG*^{KI} knock in strain via TRACE.

The heterozygous diploid strains were generated via cell fusion by coculturing a NAT marked mating type α strain and a NEO marked mating type **a** strain on V8 agar plate for 12 hours. The mixture was then collected, spread onto YPD plates with nourseothricin + G418 double selective drugs and incubated at 37℃ for three days to select the fusion products. The colonies appeared on the double selective drug plate were confirmed by diagnostic PCR and their ploidy was confirmed by flow cytometry.

All the plasmids used in this study are listed in Table S2. The primers used for plasmids and strains construction and confirmation were listed in Table S3.

Chromatin Immunoprecipitation (ChIP)

ChIP assay was performed as previously described (10). Briefly, the overnight culture of the P_{GPD1}-*MSD1-3HA* and XL280 strains were spotted onto YPD plate and incubate at 30℃ for 24h. The cells were then collected and fixed in 1xPBS buffer containing 1% formaldehyde at 30˚C for 10 min with shaking, and cross-linking was quenched by adding glycine to a final concentration of 125 mM and incubate for 5 min. The cells were then broken for five times with ChIP lysis buffer (50 mM HEPES pH 7.5, 150 mM NaCl, 1 mM EDTA, 0.5% Triton X-100, 0.1% sodium deoxycholate, 0.1% SDS, 1 mM of PMSF, and proteinase inhibitor cocktail) under a homogenizer (Precellys Evolution, Bertin, France). The crude cell lysates were subjected to sonication using an Ultrasonic Cell Disruptor (XM-2026A, Xiaomei Ultrasonic Instrument Co., LTD) to achieve an average DNA fragment size ranging from approximately 200~500 bp. Following ultrasonication, the samples were centrifuged at 20,000 g and 4 °C for 15 minutes to obtain the supernatant, and the protein concentration was determined by using a BCA protein assay kit (A65453, Thermo Fisher). Samples were subsequently adjusted to a final concentration of 2 mg/mL. An aliquot of 100 µL from each diluted sample was saved as Input. Immunoprecipitation was then performed by adding 30 µL of anti-HA nanobody agarose beads (KTSM1305, KT Life technology, Shenzhen, China) to 1 mL the diluted cell lysates and gently shaking at 4˚C for 5 h. After incubation, the beads were washed once with 1 mL ChIP lysis buffer, 1 mL high salt wash buffer (ChIP lysis buffer plus 0.5 M NaCl), 1 mL LNDET buffer (10 mM Tris-HCl pH 8.0, 0.25 M LiCl, 0.5% NP-40, 0.5% sodium deoxycholate, 1 mM EDTA), 1 mL TE buffer (10 mM Tris-HCl pH 8.0, 1 mM EDTA) in the rotator at 4˚C for 5 min respectively. Following immunoprecipitation and washes, the DNA was eluted using 500 µL of elution buffer (10 mM Tris-HCl, pH 8.0, 1 mM EDTA, 1% SDS) at 65°C for 15 minutes, followed by treatment with RNase A at 45°C for 1 hour and subsequently with proteinase K under the same conditions. The DNA was purified using phenol-chloroform extraction method, followed by ethanol precipitation and subsequently resuspended in 50 µL of nuclease-free water. Real-time quantitative PCR was conducted with both input and immunoprecipitated chromatin DNA

samples using the ABI RT-qPCR system (ABI QuantStudio Dx). All primers used for ChIP-qPCR were listed in Table S3. The relative enrichment of DNA fragments was determined as a percentage of input DNA (Input%) based on RT-qPCR analysis, as previously described (10).

SI References:

1. Lin X, Huang JC, Mitchell TG, & Heitman J (2006) Virulence attributes and hyphal growth of C. neoformans are quantitative traits and the MATalpha allele enhances filamentation. *PLoS genetics* 2(11):e187.
2. Zhai B*, et al.* (2013) Congenic strains of the filamentous form of Cryptococcus neoformans for studies of fungal morphogenesis and virulence. *Infection and immunity* 81(7):2626-2637.
3. Nielsen K*, et al.* (2003) Sexual cycle of Cryptococcus neoformans var. grubii and virulence of congenic a and alpha isolates. *Infection and immunity* 71(9):4831-4841.
4. Fraser JA*, et al.* (2005) Same-sex mating and the origin of the Vancouver Island *Cryptococcus gattii* outbreak. *Nature* 437(7063):1360-1364.
5. Wang L*, et al.* (2014) Morphotype transition and sexual reproduction are genetically associated in a ubiquitous environmental pathogen. *PLoS Pathog* 10(6):e1004185.
6. Cao Y*, et al.* (2017) Rce1, a novel transcriptional repressor, regulates cellulase gene expression by antagonizing the transactivator Xyr1 in *Trichoderma reesei*. *Mol. Microbiol.* 105(1):65-83.
7. Wang L, Zhai B, & Lin X (2012) The link between morphotype transition and virulence in *Cryptococcus neoformans*. *PLoS Pathog* 8(6):e1002765.
8. Fan Y & Lin X (2018) Multiple Applications of a Transient CRISPR-Cas9 Coupled with Electroporation (TRACE) System in the *Cryptococcus neoformans* Species Complex. *Genetics* 208(4):1357-1372.
9. Fan Y & Lin X (2020) An intergenic "safe haven" region in *Cryptococcus neoformans* serotype D genomes. *Fungal Genet Biol* 144:103464.
10. Lin J*, et al.* (2019) Transcription factor Znf2 coordinates with the chromatin remodeling SWI/SNF complex to regulate cryptococcal cellular differentiation. *Commun. Biol.* 2:412.
